# Physiological arousal as a predominant source of individual differences in functional brain networks

**DOI:** 10.1038/s42003-026-10624-y

**Published:** 2026-07-10

**Authors:** Justine A. Hill, Tianye Zhai, Cole Korponay, Julia C. Welsh, Betty Jo Salmeron, Thomas J. Ross, Yihong Yang, Blaise deB. Frederick, Amy C. Janes

**Affiliations:** 1 National Institute on Drug Abuse Intramural Research Program, Baltimore, MD USA; 2 McLean Hospital, Belmont, MA USA; 3 Harvard Medical School, Boston, MA USA

**Keywords:** Cognitive neuroscience, Neuroscience, Biomarkers

## Abstract

Characterizing individual variation in brain network organization is essential for developing fMRI-based biomarkers in precision psychiatry. We show that arousal, assessed by systemic low-frequency oscillation (sLFO) amplitude in the fMRI signal, is strongly associated with interindividual variability in functional brain network properties (e.g., R = 0.70 for default mode network (DMN)–dorsal attention network (DAN) connectivity; R = −0.63 for DMN dynamics), with replication across independent sessions and samples. These relationships persist after regressing the sLFO out of the BOLD signal and are replicated using established physiological arousal indices, indicating that arousal-related physiology significantly contributes to variability in brain network measures. Pharmacological manipulation shows that increased arousal under catecholaminergic agonism is associated with enhanced network differentiation, whereas antagonism produces the opposite effect. These findings highlight arousal-related physiological processes as a major contributor to individual differences in functional brain network properties as estimated with fMRI and indicate that catecholaminergic modulation is associated with these effects via arousal-related processes.

## Abstract (web summary)

Individual differences in arousal, indexed by a non-neuronal fMRI signal, are strongly associated with variation in functional brain network connectivity. Effects replicate within and across samples and are altered by catecholaminergic manipulation.

## Introduction

Spontaneous fluctuations in brain activity are thought to underlie functional network organization and are widely interpreted as reflecting regionally specific neuronal communication. These fluctuations give rise to stable patterns of functional connectivity that vary across individuals and are increasingly used to define network-level markers of cognition and psychiatric illness. However, emerging evidence suggests that a portion of this intrinsic variability may be driven not solely by local neuronal interactions, but by global physiological arousal. Predictive modeling in rodents demonstrates that physiological arousal alone can explain a large fraction of this spontaneous brain activity^1^, and converging human neuroimaging studies suggest that arousal-related processes may contribute to large-scale patterns observed in functional connectivity estimates^2–9^. Consistent with this perspective, the present study focuses on quantifying the contribution of arousal-related physiological processes on functional connectivity and temporal dynamics.

Early functional magnetic resonance imaging (fMRI) research identified specific neural signatures associated with arousal^10,11^. More recent work, however, indicates that physiological arousal is closely intertwined with large-scale functional brain networks, which were initially identified *independently* from systemic physiology. Specifically, evidence indicates that arousal is related to canonical brain networks, which are composed of spatially discrete brain regions that reliably co-activate to support specific cognitive, affective, or sensory functions and whose dysfunction is implicated across neuropsychiatric conditions^2,4,7,9,12–15^. For example, the Default Mode Network (DMN), involved in internally oriented attention, shows activation that precedes pupil dilation (a reliable marker of arousal) and this DMN activity in turn predicts suppression of the sensorimotor network (SMN)^15^. Others have shown that increased arousal is associated with DMN suppression and activation of a “Task Positive Network”^2^. Task Positive Networks include the SMN as well as the Salience Network (SN) and Dorsal Attention Network (DAN), which have been traditionally characterized as “task positive” because they support externally oriented attention, which is typically required in most neuroimaging tasks. Collectively, these findings suggest that arousal is associated with changes in the balance between large-scale network activity patterns, resulting in the strengthening of antagonistic interactions between the DMN and externally oriented networks^3,5^. This interpretation is consistent with neurochemical models of arousal, in which increased catecholaminergic function increases neural gain, amplifying dominant activity patterns and attenuating competing signals^16,17^. By increasing the contrast between competing large-scale systems, such processes may reduce cross-network interference and promote stronger functional network differentiation, irrespective of which network is momentarily active.

Despite this, the field of functional imaging often disregards the primary role that arousal-related processes may have in functional brain network investigations. A primary reason for disregarding arousal in fMRI neuroimaging studies is methodological constraints. It is challenging, and can be costly, to measure arousal independently of the fMRI scan. Further, even if arousal is measured, it is always measured somewhat by proxy, as precisely defining “arousal” has long been a challenge in the field. This is because arousal is a multidimensional construct encompassing both physiological and psychological components. Physiological arousal reflects changes in bodily systems (e.g., heart rate, pupil diameter, respiration, blood flow), while psychological arousal captures subjective alertness or attentional engagement^18,19^. Although related, these components can diverge depending on context or individual differences. As such, measures of arousal often capture only a subset of the underlying processes and are susceptible to extraneous influences.

Here, we address both the challenge of measuring arousal in fMRI studies and the challenge of capturing a more central index of arousal by leveraging scientific advancements in our understanding of the distinct components of the BOLD signal. Specifically, we use the BOLD signal to concurrently measure systemic physiology in addition to the more conventional measurement of neuronal activation. One distinct component of the BOLD signal is the systemic low-frequency oscillations (sLFO)^20^. The sLFO reflects slow fluctuations in blood oxygenation and volume, typically in the 0.009–0.15 Hz range, which can be measured in the brain and peripherally (for example, with pulse oximetry on a fingertip). These sLFO signals at distinct locations in the body are highly correlated, differing primarily in relative delay due to blood arrival times^20,21^. Importantly, the sLFO is physiologically distinct from task-evoked and resting state BOLD signals, which occur in the same frequency band, and are commonly interpreted as reflecting focal neuronal activity because they are driven by local neurovascular coupling. As a result, the sLFO has traditionally been treated only as “physiological noise.” However, the sLFO itself carries meaningful information about autonomic function. Recent work demonstrates that the dynamic sLFO amplitude co-fluctuates with multiple indices of physiological arousal, including smooth muscle contractions, respiration, and pupil diameter^22^. This comprehensive investigation of the sLFO found that the sLFO is positioned to operate as a window into the function of the autonomic nervous system. Further, an sLFO-derived metric—the mean sLFO amplitude—correlates strongly with several traditional indices of arousal (at *R* > 0.90 for each physiological index)^23^. These relationships collectively indicate that higher arousal is strongly associated with lower sLFO amplitude. Notably, given that the sLFO reflects a global, systemic component of the BOLD signal, it can contribute to physiological noise in functional connectivity, including systematic inflation of functional connectivity estimates, if not explicitly accounted for^23^. Accordingly, in the present study, we not only use the sLFO for insight into autonomic arousal state, but we also estimate the sLFO to reduce its contribution to the BOLD signal using a targeted, time-delay–informed regression approach prior to estimating functional connectivity. This allowed us to examine the relationship between sLFO-derived arousal and functional connectivity measures with reduced influence of global, arousal-linked systemic signal components that may otherwise contribute to large-scale correlations^23^. Equipped with this fMRI-derived arousal measure, we conducted a large-scale study to examine how sLFO-assessed arousal relates to patterns of functional connectivity (network-network correlations) and the time-varying activity of functional brain networks. Because the mean sLFO amplitude has only been recently identified as an index of arousal, we also replicated relationships between arousal and network properties using more traditional indices of arousal. We first assessed relationships between the mean sLFO amplitude and network-network connectivity between all pairs of seven canonical brain networks. Given its predominant role at rest and known interactions with externally oriented networks, we then focused on the DMN. Based on prior work, we hypothesized that heightened arousal (lower sLFO) would be associated with stronger anti-correlations between the DMN and externally oriented networks. Next, we assessed how arousal is related to the time-varying activity of individual brain networks. Such dynamic network analyses capture fluctuations in network activity across the scan, providing insights into moment-to-moment brain states which demonstrate links to specific facets of cognition and psychopathology^24–29^, and build on static understandings of brain organization^30–32^.

Finally, we examined these relationships following administration of methylphenidate and haloperidol, which are catecholaminergic agonists and antagonists, respectively. This pharmacological manipulation allowed us to test whether the relationship between arousal and network properties was sensitive to, or perhaps driven by, dopamine/norepinephrine signaling. Indeed, both dopamine and norepinephrine are deeply involved in regulating arousal, including modulating arousal-related changes in neural gain^33–37^, and they have been shown to modulate functional network properties^38–41^. Collectively, the present study (1) equips the field with an fMRI-derived measure of arousal, (2) characterizes how arousal is associated with variations in functional network differentiation and time spent in cognitive network states, and (3) provides insight into the role of catecholaminergic signaling in this brain-body relationship. Given that arousal-brain network relationships are not fully understood^42^, this work provides an important framework to refine brain-based models of cognition and disease.

## Results

### Results overview

**Fig. 1:**
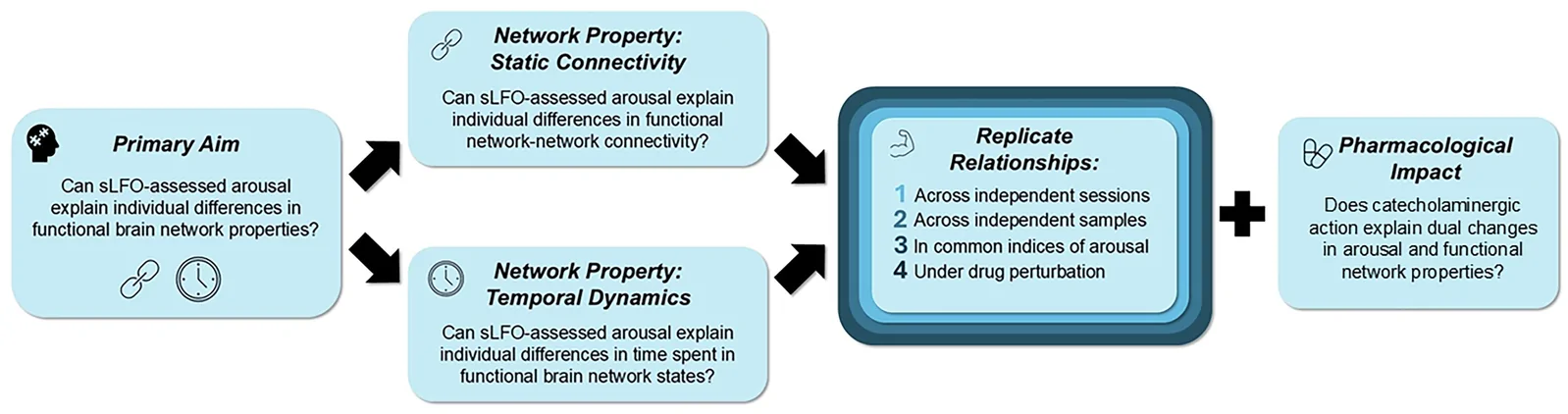
Overview of the present work. The first aim of the present work is to understand whether arousal, measured as mean sLFO amplitude, may explain individual differences in functional brain network properties. Functional network properties investigated include both static network-network connectivity and time in functional network states. Relationships were replicated across multiple metrics. The second aim of the work was to test whether catecholaminergic signaling drives dual changes in arousal and functional network properties.

An overview of the present work is depicted in Fig. 1. In all analyses, we use the mean sLFO amplitude averaged over the brain as our primary sLFO measure, as this metric serves as an index of arousal^23^. For brevity, we refer to the mean sLFO amplitude as “mean sLFO” or “sLFO.”

### Mean sLFO × functional network-network connectivity

**Fig. 2:**
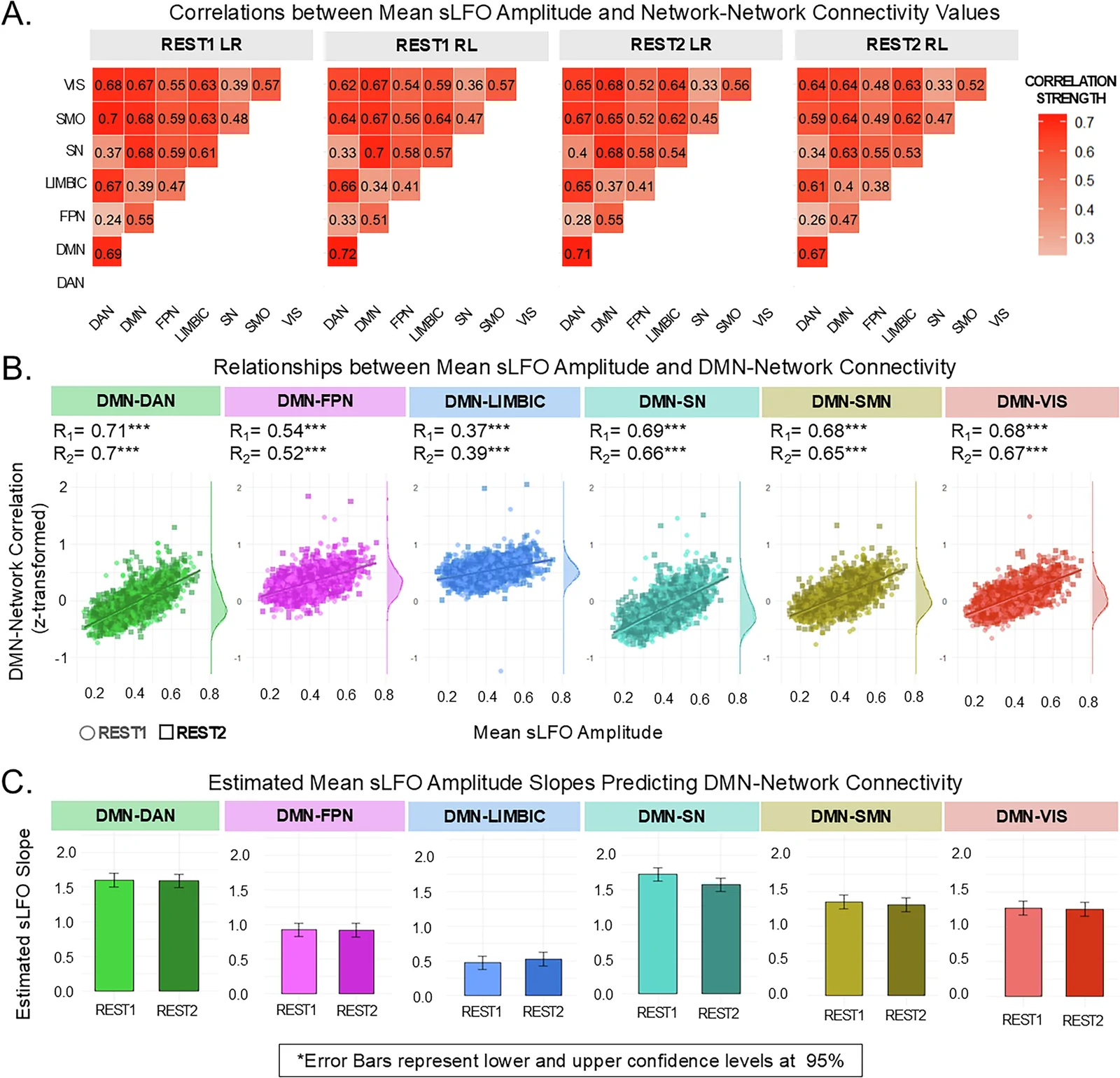
Mean sLFO amplitude explains a large proportion of individual differences in network-network connectivity. **A** Correlations between mean sLFO amplitude and functional network-network connectivity values. **B** Relationships between mean sLFO amplitude and DMN-network connectivity. Pearson’s correlation coefficient for session 1 is indicated as R_1_, and session 2 is indicated as R_2_. **C** Estimated mean sLFO amplitude slopes predicting DMN-Network activity. Each bar represents a single estimated sLFO slope derived from the combined data of all subjects (instead of a mean value). The error bars indicate the corresponding confidence intervals around that estimate. Models controlled for age, sex, phase encode direction, and within-subject random effects of *n* = 462 individuals from the HCP. DMN Default Mode Network, DAN Dorsal Attention Network, FPN Frontoparietal Network, LIMBIC Limbic network, SN Salience Network, SMN Somatomotor Network, VIS Visual Network. ***p < 0.001.

Analyses were conducted with data from the Human Connectome Young Adults Project (HCP-YA), which consists of two scan sessions of data per subject. We first confirmed that sLFO denoising reduced apparent global functional connectivity (Supplementary Fig. S1). After this confirmation, we began our analysis by testing whether the mean sLFO (i.e., the fraction of the LFO band variance in each voxel explained by the voxel-wise sLFO regressor, averaged over the brain) was broadly related to functional network-network interactions. We tested 21 linear models relating the mean sLFO to Fisher r-to-z transformed network-network connectivity of each network-network interaction across 7 canonical brain networks, controlling for age, sex, phase encode direction, and scan session. Mean sLFO was significantly related to each network-network connectivity pair after Bonferroni correction for 21 comparisons (*p* < 0.001; Supplementary Table S1; Fig. 2A).

We then focused on how the DMN, the predominant functional brain network at rest, was communicating with other functional brain networks. We conducted a mixed linear model to test the relationship between mean sLFO and connectivity between the DMN and the following remaining six canonical brain networks: DMN-DAN, DMN-Frontoparietal Network (FPN), DMN-SN, DMN-Limbic Network, DMN-SMN, and DMN-Visual Network (VIS). Models controlled for age, sex, phase encode direction (AP vs. PA), and session (REST1 and REST2). There were main effects of mean sLFO (*F*_(1/8118.1)_ = 4711.57, *p* < 0.001) and network (*F*_(5/2298.41)_ = 2487.43, *p* < 0.001). There was also a significant mean sLFO × network interaction (*F*_(5/7857.16)_ = 164.72, *p* < 0.001), indicating network-specific relationships between the sLFO and DMN-network connectivity (Supplementary Fig. 2B; Table S2). There were also main effects of sex (*F*_(1/460.93)_ = 4.27, *p* = 0.04) and session (*F*_(1/8306.5)_ = 8.32, *p* = 0.004), indicating an overall effect of these variables on network connectivity values. There was no main effect of phase encode direction (*p* > 0.05).

All sLFO × DMN-network relationships exhibited the same directionality, such that lower sLFO (indexing heightened arousal) was related to stronger DMN-network anti-correlation (Fig. 2B). Thus, to conduct a post-hoc analysis of the sLFO × DMN-network interaction—namely, differences in the strength of association across the six DMN-network pairs—we tested pair-wise comparisons of the sLFO slopes predicting network connectivity across each DMN-Network (Fig. 2C; Table S3). In session 1, this post-hoc analysis revealed that there was no significant difference in the sLFO slope predicting DMN-DAN correlation compared to the sLFO slope predicting DMN-SN correlation (*z* = 2.032, *p* = 0.32). There was also no significant difference in the sLFO slope predicting DMN-SMN connectivity compared to the sLFO slope predicting DMN-VIS connectivity (*z* = 1.38, *p* = 0.93). All other pair-wise comparisons of sLFO slopes significantly differed from each other when correcting for multiple comparisons (*p* < 0.003), such that DMN-DAN and DMN-SN sLFO slopes were significantly greater than all other networks, and DMN-SMN and DMN-VIS sLFO slopes were significantly greater than DMN-FPN and DMN-Limbic sLFO slopes. These findings were precisely replicated in session 2 data (Table S3). These results indicate that the strength of relationships between (1) sLFO and DMN-DAN connectivity and (2) sLFO and DMN-SN connectivity do not differ from one another and are significantly greater in magnitude than all other sLFO × DMN-network relationships.

### Reproducibility of mean sLFO × functional network-network connectivity

To determine reproducibility (1) within the HCP-YA sample across separate scan days and (2) across independent samples (Replication sample *N* = 59), we conducted Bayes factor analysis. In contrast to testing a linear model for an sLFO × session or sample interaction, which is meant to test for significant *differences* (and thus is not suited to test or quantify *sameness*), Bayes factor analysis allows us to quantify the likelihood of sameness (i.e., reproducibility) or difference in the relationships between sLFO and brain network organization across sessions or samples.

Each Bayes factor was computed for each specific DMN-network (DMN-DAN, DMN-Limbic, etc.). For the first aim, testing reproducibility across sessions, we compared a null model (session, phase, age, sex, within-subject covariates) relating network-network connectivity to mean sLFO to an alternate model which included a session x mean sLFO interaction. Based on interpretation from Lee and Wagenmakers^43^, Bayes factor analysis supported session reproducibility for all sLFO × DMN-network relationships at moderate-very strong levels (Table 1).

**Table 1:** Relationship between DMN-network connectivity and mean sLFO is reproducible in independent sessions and independent samples

Connectivity	Analysis Bayes factor (H0/HA)	
Between-network connectivity	Between sessions, within the HCP-YA sample	Between samples HCP-YA and *N* = 59 healthy adults
DMN-DAN	41.54 ± 9.69%	5.41 ± 10.32%
DMN-FPN	35.3 ± 8.73%	5.845 ± 6.45%
DMN-LIMBIC	6.62 ± 14.08%	5.18 ± 16.55%
DMN-SN	3.8 ± 10.85%	9.07 ± 7.61%
DMN-SMN	9.14 ± 8.63%	1.65 ± 7.99%
DMN-VIS	38.92 ± 19.26%	1.85 ± 11.13%

*DMN* Default Mode Network, *DAN* Dorsal Attention Network, *FPN* Frontoparietal Network, *LIMBIC* Limbic network, *SN* Salience Network, *SMN* Somatomotor Network, *VIS* Visual Network.

For the second aim, testing reproducibility between independent samples, we compared a complete null model (sample, age, sex, within-subject covariates) relating network-network connectivity to mean sLFO to an alternate model which included a sample × mean sLFO interaction. Bayes factor analysis supported sample reproducibility for all sLFO × DMN-network relationships at anecdotal-moderate levels (Table 1).

### Mean sLFO × time in functional network states

**Fig. 3:**
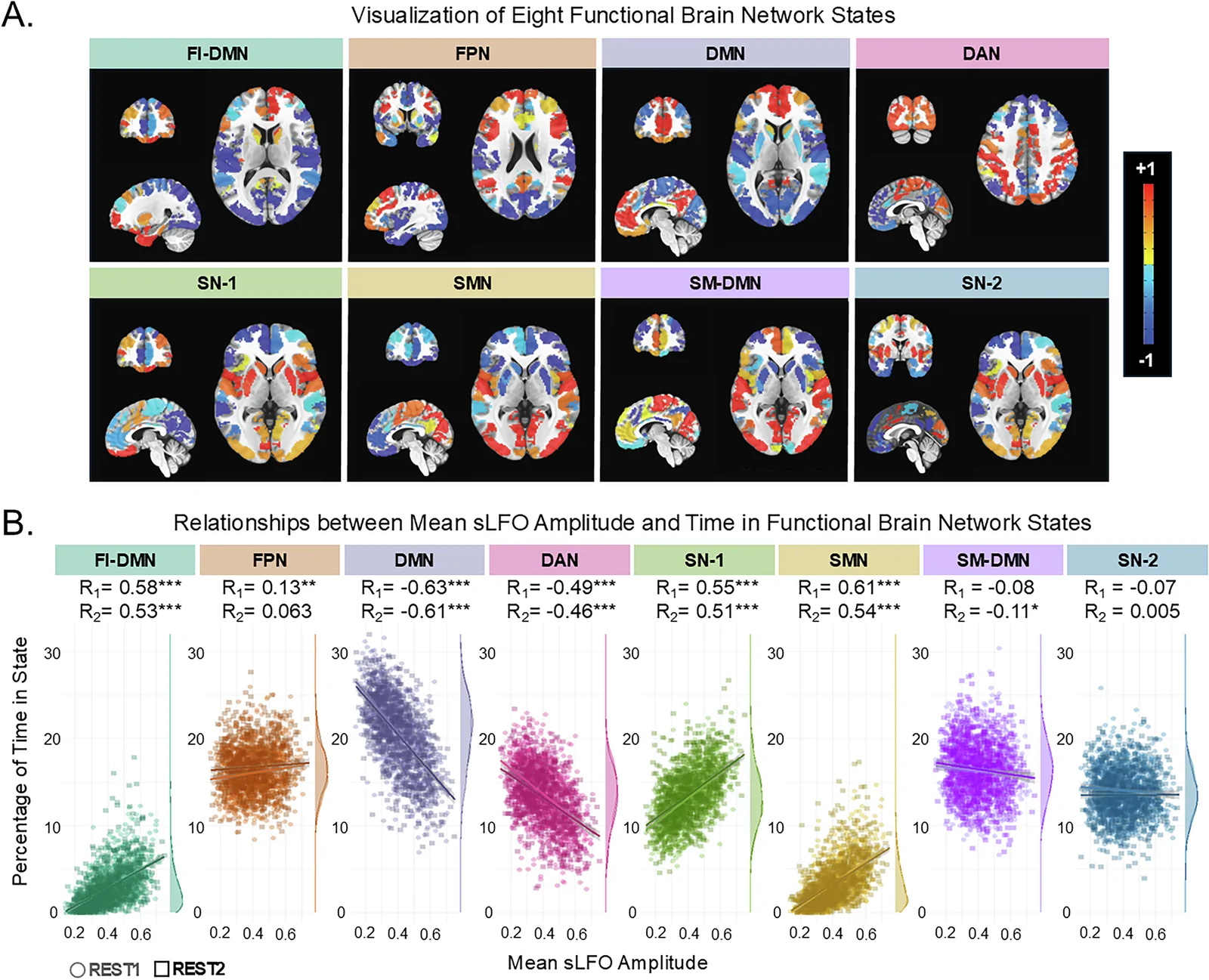
Mean sLFO amplitude explains a large proportion of individual differences in percent of time spent in network states in *n* = 462 individuals. **A** Visualization of eight functional brain network states. **B** Relationships between mean sLFO amplitude and time in functional brain network states. Pearson’s correlation coefficient session 1 is indicated as R_1_, and session 2 is indicated as R_2_. FI-DMN Frontoinsular-Default Mode Network State, FPN Frontoparietal Network State, DMN Default Mode Network State, DAN Dorsal Attention Network State, SN-1 Salience Network State 1, SMN Somatomotor Network State, SM-DMN Somatomotor-Default Mode Network State, SN-2 Salience Network State 2. *p < 0.05, **p < 0.01, ***p < 0.001.

We conducted a mixed linear model to test the relationship between mean sLFO and percent of time spent in brain network state across the eight brain network states previously derived via CAP analysis in this large sample of healthy young adults^44^. The brain network states, which align with known functional brain networks, are as follows: frontoinsular-DMN (FI-DMN), FPN, DMN, DAN, SN-1, sensorimotor occipital (SMN), sensorimotor-DMN (SM-DMN), and SN-2 (Fig. 3A).

There was a main effect of brain state (*F*_(7/6493.48)_ = 2101.1, *p* < 0.001) and a significant interaction between sLFO and brain state when relating to percentage of time in state (*F*_(7/14141.11)_ = 508.4, *p* < 0.001). There were also significant brain state × sex (*F*_(8/3701.63)_ = 9.44, *p* < 0.001), brain state x age (*F*_(8/3691.93)_ = 13.61, *p* < 0.001), and brain state × session interactions (*F*_(8/11063.7)_ = 13.72, *p* < 0.001) (Fig. 3B; Table S4).

### Reproducibility of mean sLFO × time in functional network states

Mirroring the approach used for testing reproducibility between mean sLFO and DMN-network connectivity, we used Bayes factor analysis to test for reproducibility in the relationships between sLFO and brain network temporal dynamics. In testing reproducibility between sessions, Bayes factor analysis revealed anecdotal-very strong evidence of between-session reproducibility for all brain network states (Table 2). For testing reproducibility between samples, Bayes factor analysis supported sample reproducibility for the DMN, DAN, SM-DMN, and SN-2 at anecdotal-moderate levels, while in contrast, there was very strong evidence of a sample effect for the FI-DMN and FPN. For SN-1 and SMN, the level of evidence neared 1 (0.86, 0.91; respectively), suggesting no evidence for either a sample effect or between-sample reproducibility (Table 2).

**Table 2:** The relationship between the percentage of time spent in functional network states and the mean sLFO is reproducible in independent sessions and samples in a network-specific manner

Dynamics	Analysis Bayes factor (H0/HA)	
Brain network state	Between sessions, within the HCP-YA sample	Between samples HCP-YA and *N* = 59 healthy adults
FI-DMN	7.66 ± 8.56%	0.006 ± 7.24%
FPN	2.07 ± 3.14%	0.0054 ± 3.82%
DMN	6.18 ± 5.96%	3.72 ± 4.98%
DAN	11.3 ± 12.72%	3.32 ± 6.89%
SN-1	24.5 ± 9.91%	0.86 ± 11.38%
SMN	50.75 ± 20.18%	0.91 ± 10.46%
SM-DMN	5.57 ± 3.89%	7.87 ± 3.34%
SN-2	3.17 ± 2.63%	3.97 ± 2.76%

Between Sessions: Bayes Factor analysis reveals that the relationship between sLFO and FI-DMN, DMN, DAN, SN-1, SMN, SM-DMN, and SN-2 is reproducible across sessions. Between Samples: Bayes Factor analysis reveals that the relationship between percentage of time spent in functional network states and mean sLFO is reproducible in an independent sample of healthy adults for DMN, DAN, SM-DMN and SN-2. For FI-DMN and FPN, Bayes Factor analysis reveals that a sample effect is supported. For SN-1 and SMN, an anecdotal sample effect nearing 1 (no evidence) is supported.

*FI-DMN* Frontoinsular Default Mode Network State, *FPN* Frontoparietal Network State, *DMN* Default Mode Network State, *DAN* Dorsal Attention Network State, *SN-1* Salience Network State 1, *SMN* Somatomotor Network State, *SM-DMN* Somatomotor-Default Mode Network State, *SN-2* Salience Network State 2.

### Within-subject changes in mean sLFO and functional brain network properties

After demonstrating that individual variance in mean sLFO accounts for a large proportion of individual differences in brain network interactions and activity *between* subjects, we aimed to test whether intra-subject variance in mean sLFO was associated with variation in measures of brain function within subjects—in other words, we tested whether change in mean sLFO across the two scan sessions related to change in brain DMN-network connectivity and time in network states across the two scan sessions.

**Fig. 4:**
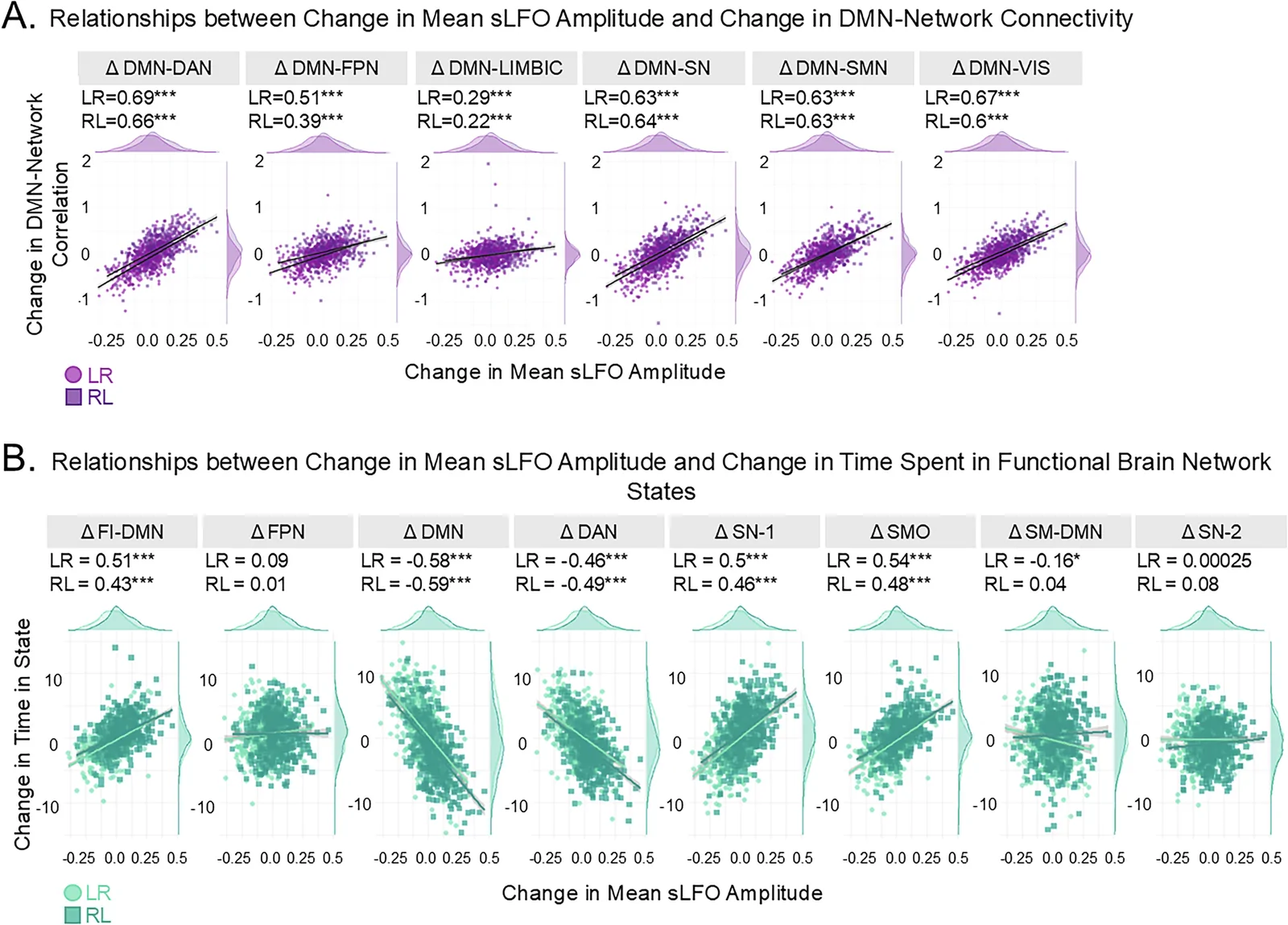
Intra-subject variability in mean sLFO amplitude explains a large proportion of intra-subject variability in network-network connectivity and time spent in network states in *n* = 462 individuals. **A** Relationship between change in mean sLFO amplitude and change in DMN-network connectivity. **B** Relationship between change in mean sLFO amplitude and change in time spent in network states. For both (**A**, **B**), change was measured within subject from scan session 1 to scan session 2 for two phase encodings (LR/RL); corresponding *R* values are noted. DMN Default Mode Network, DAN Dorsal Attention Network, FPN Frontoparietal Network, LIMBIC Limbic network, SN Salience Network, SMN Somatomotor Network, VIS Visual Network. FI-DMN Frontoinsular Default Mode Network State, SM-DMN Somatomotor-Default Mode Network State. *p < 0.05, **p < 0.01, ***p < 0.001.

For DMN-network connectivity, there was a significant effect of ΔsLFO (*F*_(1/4539.56)_ = 1947.68, *p* < 0.001) and a significant ΔsLFO × network interaction (*F*_(5/5070.35)_ = 112.64, *p* < 0.001) in relating to ΔDMN-network connectivity (Fig. 4A). There was also a significant effect of phase encoding direction (*F*_(1/5315.71)_ = 132.41, *p* < 0.001). However, in an additional model, we confirmed there was no ΔsLFO × phase encode direction interaction, indicating that the relationship between change in sLFO and change in network connectivity is consistent across phase encode directions (*p* > 0.05). Mirroring between subject findings, each ΔDMN-network relationship was positively related to ΔsLFO (Fig. 4A). The strength of correlations between ΔsLFO and ΔDMN-network connectivity varied by network in a manner that also precisely aligned with the between-subject correlations (Supplementary Fig. S2; Table S5).

For network dynamics, in the main model relating to Δtime in state, there was a significant effect of brain state (*F*_(7/14744)_ = 6.97, *p* < 0.001) and there was a significant ΔsLFO × brain state interaction (*F*_(7/14744)_ = 460.58, *p* < 0.001). There were also brain state × sex (*F*_(8/14744)_ = 3.02, *p* = 0.002) and brain state × phase encode direction interactions (*F*_(8/14744)_ = 25.09, *p* < 0.001). Similarly to static findings, the relationships between change in sLFO and change in time in functional network states across scan days largely mirrored the between-subject findings (Fig. 4B; Table S6).

### Confirming relationships between functional brain networks and physiology

Having determined robust relationships between the mean sLFO—our index of systemic physiological arousal—and functional brain network properties, we sought to confirm that the relationships between mean sLFO and (1) DMN-network connectivity and (2) time in functional network states aligned with physiology x brain relationships using more traditional indices of arousal available in our dataset: respiration volume per unit time (RVT) and percent of heart rate variability (HRV) in high frequency range (HRV-HF) and low frequency range (HRV-LF).

There was a main effect of RVT on DMN-network connectivity (*F*_(1/7915.26)_ = 136.24, *p* < 0.001) and a significant RVT x network interaction (*F*_(5/7360.72)_ = 9.4, *p* < 0.001). There was a main effect of HRV-LF on DMN-network connectivity (*F*_(1/7840.69)_ = 182.56, *p* < 0.001) and an HRV-LF × DMN-network interaction (*F*_(5/6352.69)_ = 7.6, *p* < 0.001). There was also a main effect of HRV-HF on DMN-network connectivity (*F*_(1/7513.89)_ = 23.31, *p* < 0.001); however, there was no HRV-HF × network interaction, indicating a non-network-specific relationship between HF-HRV and DMN-network connectivity (*p* > 0.05). The directionality of the relationships is as follows: RVT and HRV-HF are negatively related to DMN-network connectivity; HRV-LF is positively related to DMN-network connectivity. Thus, these findings confirm that, as measured by the mean sLFO and as consistent with the literature^5^, increased arousal (now indexed as higher RVT and HRV-HF and lower HRV-LF) is related to stronger DMN-network anti-correlation. Post-hoc analysis also confirmed that significant network-specific differences in the strength of the alternate indices of arousal predicting DMN-network connectivity, which were tested/detected with these indices aligned with the network-specific differences in the strength of the sLFO slopes predicting DMN-network connectivity (Fig. S3A; Tables S7 and S8).

In testing relationships between conventional measures of arousal and time in functional network states, the following interactions were detected in three separate models: brain state × RVT (*F*_(7/13456.6)_ = 23.76, *p* < 0.001), brain state x HRV-LF (*F*_(7/13196.18)_ = 32.64, *p* < 0.001), brain state × HRV-HF (*F*_(7/12246.98)_ = 4.88, *p* < 0.001). Network-specific relationships between alternate indices of arousal and time in network states again aligned with sLFO relationships in both strength and directionality (Fig. S3B; Table S9). Finally, we tested whether within-subject changes in RVT, HRV-HF, and HRV-LF mirrored our findings of relationships between within-subject changes in sLFO and changes in network properties. See supplement for results (Fig. S3).

### Mean sLFO, functional brain networks, and catecholaminergic pharmacology

After determining relationships between the mean sLFO and functional brain network properties in a sample of healthy adults under homeostatic conditions, we inquired whether such relationships would remain stable in healthy adults under pharmacological perturbation. This sample underwent three fMRI scan sessions, on separate days, under the following drug conditions: placebo, methylphenidate, and haloperidol (in a double-blind, randomized fashion).

For static network-network connectivity, in a linear model which controlled for age and sex, there were main effects of sLFO (*F*_(1/668.94)_ = 119.45, *p* < 0.001), network (*F*_(5/321.60)_ = 173.07, *p* < 0.001), drug (*F*_(2/695.48)_ = 4.17, *p* = 0.016), and age (*F*_(1/56.17)_ = 5.65, *p* = 0.021) on DMN-network connectivity. There were no significant sLFO × drug (*p* > 0.05) or network × drug (*p* > 0.05) interactions. There was no significant drug × sLFO × network interaction, indicating that the drug does not significantly alter the relationship between mean sLFO and DMN-network connectivity (*p* > 0.05; Supplementary Tables S10 and S11).

For time in functional network states, in a linear model which controlled for age and sex, there was a main effect of brain state (*F*_(7/479.76)_ = 296.73, *p* < 0.001) and there were interactions of sLFO × brain state (*F*_(7/1235.96)_ = 28.88, *p* < 0.001), drug x brain state (*F*_(14/970.60)_ = 3.74, *p* < 0.001) and age × brain state (*F*_(8/443.90)_ = 1.97, *p* = 0.049) on percent time in state. Again, there was no three-way sLFO × brain state x drug interaction, indicating that the mean sLFO × time in network state relationship remains consistent across pharmacological perturbation (*F*_(14/1174.73)_ = 1.48, *p* > 0.05; Supplementary Tables S12 and S13).

Having determined that the relationships between sLFO and brain network organization are not significantly modulated by drug, we set out to test whether catecholamine manipulation may drive changes in arousal and functional network properties. Our pharmacological manipulation allowed us to probe mean sLFO × brain network relationships under drug conditions with antagonistic effects on both arousal and catecholamines: methylphenidate is a psychostimulant and a dopamine and norepinephrine agonist (through blocking reuptake of dopamine and norepinephrine), and haloperidol has sedative properties and is a dopaminergic D2 receptor antagonist. For this analysis, we concentrated on the DMN, DAN, and SN. Our focus on DMN, DAN, and SN was guided by both methodological and scientific rationales. From a methodological standpoint, our HCP-YA sample showed that relationships between the sLFO and DMN, DAN, and SN were often the strongest relative to other networks and were largely reproducible, including existing in the pharmacological manipulation sample (Fig. S4). From a scientific perspective, there is robust evidence that the DMN, DAN, and SN (and their interactions) hold catecholamine-related functional and clinical relevance^38,45–51^. As such, we were interested in how changes in (1) DMN-DAN and DMN-SN connectivity and (2) time spent in the DMN, DAN, and SN under catecholaminergic drug compared to placebo relate to changes in mean sLFO under catecholaminergic drug compared to placebo.

**Fig. 5:**
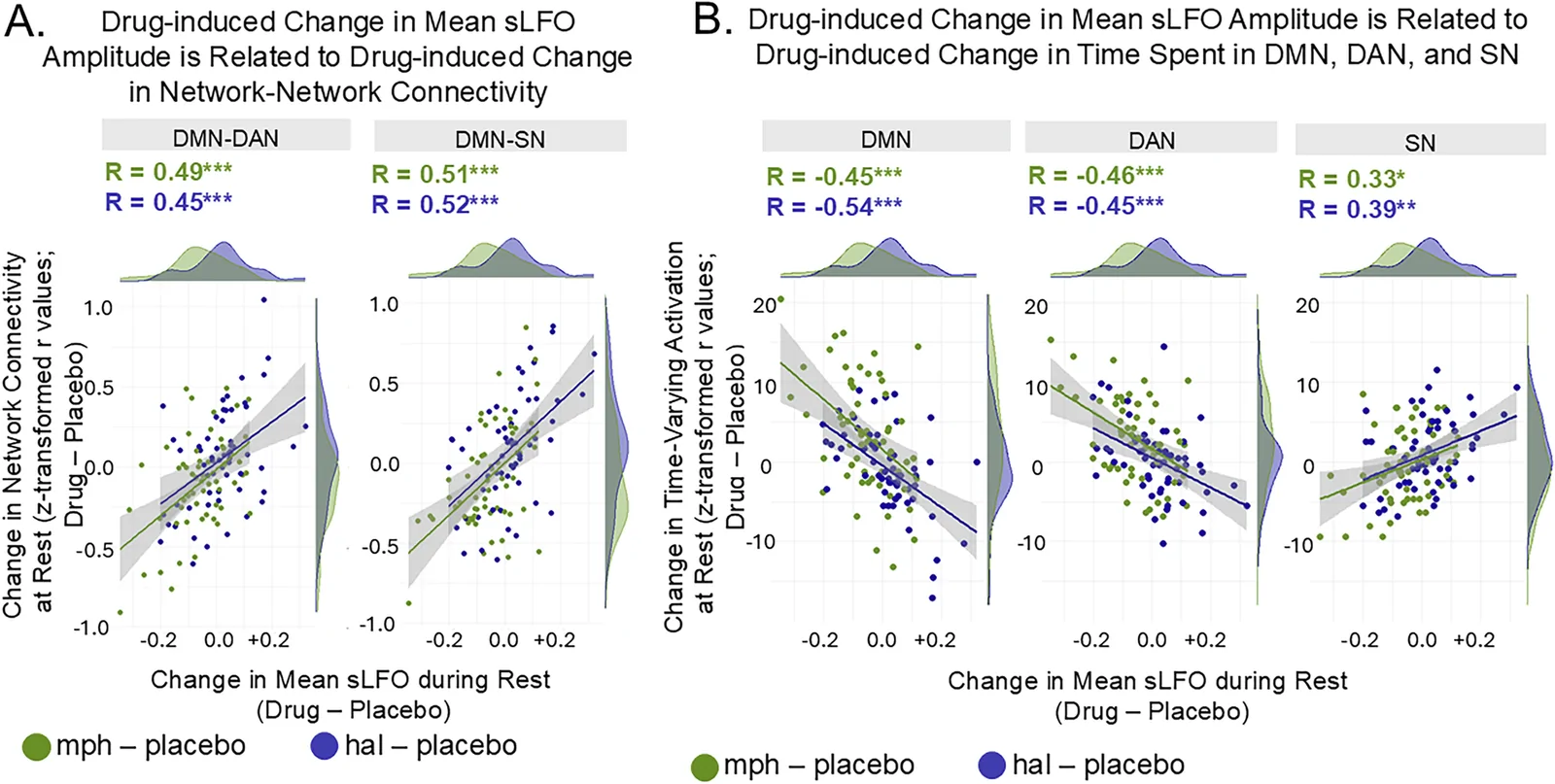
Drug-induced change in mean sLFO amplitude explains a large proportion of variability in drug-induced changes in functional network properties using a repeated measures design involving *n* = 59. **A** Drug-induced change in mean sLFO during rest is related to drug-induced impact on DMN-DAN connectivity and DMN-SN connectivity. The positive direction of the relationship indicates that drug-induced decrease in sLFO (increase in arousal) is related to strengthening of DMN-DAN and DMN-SN anti-correlation, whereas drug-induced increase in sLFO (decrease in arousal) is related to weakening of DMN-DAN and DMN-SN anti-correlation. **B** Drug-induced change in mean sLFO is related to drug-induced change in time spent in the DMN, DAN, and SN. DMN Default Mode Network, DAN Dorsal Attention Network, SN Salience Network. *p < 0.05, **p < 0.01, ***p < 0.001.

Critically, pharmacologically induced changes in sLFO amplitude predicted corresponding changes in canonical network architecture across individuals. For both methylphenidate and haloperidol, drug-induced modulation of sLFO was strongly associated with changes in DMN-DAN (methylphenidate *R* = 0.49, *p* < 0.001; haloperidol *R* = 0.45, *p* < 0.001) and DMN-SN anti-correlations (methylphenidate *R* = 0.51, *p* < 0.001; haloperidol *R* = 0.52, *p* < 0.001) (Fig. 5A). The directionality of these relationships is theory-consistent: methylphenidate-induced reductions in sLFO (reflecting increased arousal) were associated with strengthened network segregation, whereas haloperidol-induced increases in sLFO were associated with reduced segregation. These coordinated, bidirectional effects across opposing pharmacological manipulations argue against a purely mechanical shared-variance explanation and instead indicate structured, catecholamine-sensitive reconfiguration of large-scale functional networks. In addition to the static connectivity findings, we demonstrated that drug-induced changes in mean sLFO are related to drug-induced changes in the temporal dynamics of DMN, DAN, and SN—specifically changes in the time-varying dominant activation of these networks. Drug-induced decrease in sLFO (increase in arousal) was related to spending more time in DMN (methylphenidate *R* = −0.45, *p* < 0.001; haloperidol *R* = −0.54, *p* < 0.001) and DAN (methylphenidate *R* = −0.46, *p* < 0.001; haloperidol *R* = −0.45, *p* < 0.001) and was related to spending less time in SN (methylphenidate *R* = 0.33, *p* < 0.05; haloperidol *R* = 0.39, *p* < 0.01) (Fig. 5B).

Given the demonstrated relationships between SN, DMN, DAN activity and the catecholaminergic arousal system and the potential mechanism at play in these relationships^3,16,52^, we conducted further analysis to test whether activity of the SN is directly related to activity of the DMN and DAN using the larger HCP dataset and the pharmacological manipulation cohort. We confirmed that spending less time in the SN is directly related to spending more time in the DMN and DAN at rest (*p* < 0.001; Supplementary Fig. S5A) and that catecholamine-induced change in time in the SN is inversely related to change in time in the DMN and DAN (*p* < 0.001; Supplementary Fig. S5B).

## Discussion

Our findings demonstrate that individual differences in large-scale functional brain networks are strongly associated with systemic physiological arousal. By reducing the contribution of the systemic sLFO to the BOLD signal prior to estimating network properties, we substantially reduced the contribution of this global physiological component to measures of network function, thereby increasing the extent to which these measures reflect underlying neural contributions, while acknowledging that residual physiological influences may remain. Using this approach, we show that mean sLFO amplitude explains a substantial proportion of variance in both functional network-to-network connectivity and time-varying network dynamics. Experimentally induced changes in arousal via pharmacological manipulation are associated with corresponding alterations in high-order network properties, while sLFO-network relationships remain consistent across sessions, samples, and pharmacological states. Notably, arousal accounted for 30–50% of the variance in intrinsic network properties, representing a remarkably large effect. By identifying a robust and reproducible physiological contributor to variability, these findings provide critical insight for refining network-based models of cognition and psychiatric disease and underscore the importance of incorporating systemic physiology into large-scale neuroimaging research.

Previous work has established that the mean sLFO amplitude is tightly and inversely related to physiological arousal^22,23^. Extending this literature, we find that lower sLFO (higher arousal) is consistently related to heightened anti-correlation between the DMN and task-positive networks, such as the DAN and SN. This pattern aligns with prior studies showing that pupil diameter and electrophysiological measures of increased arousal are associated with greater network-network anti-correlation^5,8,17^ and greater network differentiation^4^. Importantly, all the relationships identified here exist *after* attenuating the temporal sLFO signal from the BOLD data used to define network properties. This procedure decreases the sLFO’s confounding influence on connectivity, leading to a reduction in global connectivity (Supplementary Fig. S1), and reduces the association between the sLFO and DMN time courses (Supplementary Fig. S6), indicating that the observed effects are not driven by the sLFO. In fact, reducing the influence of the sLFO in the BOLD data improves the relationship between mean sLFO amplitude and network connectivity (Supplementary Fig. S7). This suggests that these associations are not primarily explained by shared global signal contributions and are consistent with a relationship between physiological arousal and functional brain network measures, while acknowledging that residual non-neuronal influences cannot be fully excluded.

We additionally replicated the arousal-network relationships using traditional physiological indices of arousal, such as heart rate variability and respiration volume. Intriguingly, the sLFO amplitude explained more variance in network organization than other more established measures of physiological arousal. This high explanatory power likely reflects the sLFO’s role as a core autonomic signal, which couples with vascular tone, smooth muscle contractions, respiration, pupil diameter, and heart rate variability^22,23^. Collectively, these findings highlight the systemic relevance of the sLFO and underscore the importance of accounting for arousal when interpreting intrinsic functional brain organization.

Our analyses further demonstrate that interactions between higher-order cognitive networks, particularly the DMN-DAN and DMN-SN connectivity, show the *strongest* relationships with physiological arousal. We show that arousal accounts for ~43–50% of the variance in these network interactions, underscoring the magnitude of this relationship. These findings extend the literature by revealing both the strength and network-specificity of arousal-connectivity effects, which is consistent with evidence that systemic physiology is closely linked to brain regions supporting cognitive function^4^. The association between arousal and DMN-DAN/DMN-SN connectivity is consistent with the hypothesis that heightened arousal biases the brain toward different attentional modes while suppressing irrelevant activity—reflecting improved differentiation between internally oriented (DMN) and externally oriented (DAN, SN) processes^53,54^. Given that imbalances among (and interferences between) these specific networks are central to prominent models of psychopathology, including Menon’s triple network model^55^, the current findings indicate that arousal, measured via the sLFO, may be a critical but currently underappreciated factor in studies of functional networks in psychiatric disorders^53,54^.

We further demonstrate that sLFO-network relationships remain stable under pharmacological perturbation and that catecholamine-induced changes in arousal closely track corresponding changes in DMN-Network connectivity. The directionality of these effects fits with known drug effects: for methylphenidate, a catecholaminergic agonist, drug-induced decrease in sLFO (increase in arousal), is associated with strengthened anti-correlation between the DMN and both the DAN and SN. In contrast, haloperidol, a D2 antagonist with sedative properties, produced the opposite pattern. The finding that both the sLFO and network properties change in tandem, and in opposing directions under opposing pharmacological manipulations, suggests that catecholaminergic signaling is associated with coordinated changes in physiological arousal and functional network properties, further supporting a link between arousal and intrinsic network function.

Norepinephrine and dopamine are primary regulators of arousal and, via the arousal system, are known to modulate neural gain by amplifying prioritized activity while suppressing competing, non-task-related signals. Increased neural gain has been suggested to promote stronger network anti-correlations during tasks, as effective differentiation between large-scale systems requires amplification of relevant activity and suppression of non-relevant brain activity^3,17^. Our work is consistent with this framework, suggesting that catecholamines may be associated with enhanced network differentiation through arousal-linked gain modulation, lending to heightened anti-correlation, even at rest.

Beyond static connectivity, we show in a large sample of healthy adults that mean sLFO amplitude strongly relates to time-varying network dynamics at rest. First, we demonstrate that increased arousal is related to less time in the SMN. This is consistent with a large body of research concluding that catecholamines decrease noise in sensory regions under heightened arousal^52,56–58^ and extends such understandings to explain ~30% of the variance in the time-varying behavior of the SMN. We further show that heightened arousal was associated with increased time spent in attentional states, including both the DMN and the DAN, and decreased time in the SN. At first glance, the association between heightened arousal and increased DMN engagement may appear counterintuitive, given the historical association between the DMN as a “rest” or “task-negative” network. However, more recent work has shown that the DMN is critical for internal attentional processes, such as autobiographical memory and emotional processing^59,60^. As such, our findings suggest that heightened arousal is associated with increased engagement in attentional states, regardless of the attention type (internal vs. external), consistent with theoretical frameworks in which arousal has been linked to increased selectivity of brain activity across different cognitive contexts^52^.

Moreover, because the SN is closely linked to salience and arousal, it may appear surprising that increased arousal is related to less time in the SN state. However, it is critical to note that the impact of arousal on the attentional system varies by context, where the present context is at rest^52^. Further, a plausible explanation for this relationship lies in coordinated interactions among the DMN, DAN, and the SN. In the triple-network model, the SN facilitates network transitions between the DMN and DAN, enabling internal and external directed attention, respectively, while regulating their antagonistic balance^3,55^. Consistent with this interactive relationship, our post-hoc analysis confirmed that increased time in the DMN and DAN was directly associated with reduced time in the SN at rest.

Recent fMRI work further suggests that the SN does not act alone in coordinating DMN-DAN dynamics, but instead operates in concert with the catecholaminergic arousal system^3^. This fits with neuroanatomical and preclinical literature, where the anterior cingulate cortex—a main hub of the SN—provides cortical input onto the main nucleus of norepinephrine neurons, which regulate arousal levels (the locus coeruleus), to ultimately recruit catecholamine activity and direct attention^52,61^. From this perspective, our results indicate that elevated arousal *at rest* may bypass some of the need for SN-mediated monitoring, leading to amplified time in attentional states with less SN-facilitated coordination^7,9,62,63^. Though this hypothesis requires confirmation, it fits with our past work, which demonstrated that catecholaminergic agonism alone increases time spent in the DMN and DAN attentional states at rest, without increasing SN activity^38^.

Notably, we demonstrated that catecholamine-induced enhancement of time in the DMN and DAN, and corresponding reductions in SN engagement, are directly related to catecholaminergic modulation of arousal. This implies that arousal mechanisms may be involved in the catecholaminergic processes that are associated with DMN, DAN, and SN dynamics. Given that the DMN and DAN are crucial for attentional processes and commonly positioned as destination networks for SN-mediated coordination, it may be that at rest, DMN and DAN act as the dominant network states, which are selectively amplified by catecholaminergic enhancement of neural gain. To the best of our knowledge, this provides a novel framework for understanding how arousal enhances attention, and it fits with our static connectivity findings because amplification of DMN and DAN (at opposing times) has been shown to strengthen DMN-DAN anti-correlation^38,64^. Indeed, when considered alongside our static connectivity findings, these results suggest that catecholaminergic modulation of neural gain (suppression of irrelevant brain activity; amplification of relevant brain activity) may contribute to variability in static and temporal functional network properties. Though this hypothesis requires mechanistic confirmation, it fits with previously proposed gain-related models^16,17,52,58^ and may explain a substantial proportion of clinically-relevant variance in brain organization and dynamics.

Despite the strength and consistency of these findings in a large sample, several limitations warrant consideration. Two of the eight time-varying network states showed sample-specific effects, as the findings in the HCP sample did not replicate in the smaller sample. Differences in sample size, acquisition parameters, demographics, or other methodological factors may have contributed to the non-replicating effects, and future work using harmonized protocols will be important for clarifying the stability of these findings. Given the evidence of a sample effect, we did not focus on these states (FI-DMN, FPN) in the follow-up analysis and discussion of these results.

In addition, although we observe within-subject associations between changes in network properties and changes in both sLFO-derived and established physiological indices of arousal, these effects are based on only two time points and may therefore be influenced by session-to-session variability and measurement error. While the convergence across multiple arousal measures provides support for a meaningful physiological contribution, future work with repeated sampling across more time points and explicit assessments of test–retest reliability will be important for establishing the stability of these within-subject effects. Further, in the current analysis, we used the mean sLFO amplitude, a metric of sLFO correlation amplitude averaged across the entire brain. This reflects a deliberate modeling choice to index system-level arousal, where the robust and biologically interpretable relationships observed demonstrate that this aggregate measure is sufficient to capture meaningful associations between arousal and network organization. However, future work incorporating spatial and temporal variation in the sLFO may further refine and extend these findings.

Although we demonstrate robust relationships between the sLFO and network properties in healthy adults, and did not find an effect of age in this relatively young population, it remains unclear whether this relationship generalizes across the lifespan or in neuropsychiatric conditions. This question is particularly relevant given that autonomic nervous system dysfunction is often implicated in neuropsychiatric disorders^65–70^. Many studies interpret altered functional connectivity in these conditions as reflecting primary neural pathology, yet systemic physiological dysregulation may also contribute to observed network differences. By establishing mean sLFO amplitude as an fMRI-intrinsic measure of arousal, the present work provides a practical framework for directly testing the contribution of arousal to network-level alterations in clinical populations. As a first step, we demonstrate that arousal accounted for nearly 30% of the variance in medication-related changes in cognitive network properties, underscoring the potential relevance for refining brain-based models of disease and medication effects.

Finally, the present work focuses on the whole-brain mean sLFO amplitude, which provides a simple and robust summary measure of this signal. Prior work has demonstrated spatial variation in the sLFO, with larger changes observed in sensorimotor regions as arousal decreases, suggesting that region-specific estimates may further refine this metric^23,71^. However, the current study was intentionally designed to evaluate a global, system-level index of arousal that can be readily and consistently applied across datasets. While region-specific sLFO measures may offer additional specificity, their development and validation are beyond the scope of the present work.

In conclusion, our results demonstrate that systemic physiological arousal, as measured by mean sLFO amplitude, is a major contributor to individual differences in both static and dynamic functional network properties. The parallel shifts in sLFO and network organization/dynamics observed under methylphenidate and haloperidol suggest that arousal-related network changes vary systematically with catecholaminergic tone. These findings are consistent with the view that large-scale network properties, as estimated with fMRI, vary with neurochemical and physiological state. Together, this work provides a framework for integrating systemic physiology into large-scale neuroimaging studies and highlights the importance of accounting for arousal-related processes when interpreting functional connectivity and temporal dynamics. This opens new avenues for precision medicine approaches that leverage intrinsic physiological signals to predict individual brain function and responses to pharmacological interventions.

## Methods and materials

### Samples

*Human Connectome Project Young Adults* (HCP-YA) Cohort included participants from the HCP 1200 subject release. Data was used from 462 participants from the HCP-YA sample who were previously assessed, given they have no current or family history of certain psychiatric illness or substance use (181 male, 281 female; age 28.66 ± 3.65 years)^43^. Resting-state scans were captured on two independent days, with two scans per day recorded using different phase encode directions: right to left (RL) and left to right (LR). All participants provided written consent prior to participation.

*Acute Drug Administration Cohort* included 59 healthy individuals (17 male, 42 female, aged 18–55). Participants with mental and physical health diagnoses and/or medications that interfere with the BOLD signal or alter the metabolism of catecholaminergic agents were excluded. Participants were scanned in a resting-state paradigm following administration of 20 mg methylphenidate, 2 mg haloperidol or placebo–timed in accordance with drug peak–using a randomized, counterbalanced, double blind design. The study was reviewed and approved by the institutional review board of the National Institutes of Health, and all participants provided written informed consent prior to participation. All ethical regulations relevant to human research participants were followed. Data are available from the corresponding author (or other sources, as applicable) on reasonable request

### fMRI acquisition

#### HCP-YA resting-state scan

Details of HCP-YA scan and open-source preprocessed data can be found elsewhere^70,72^. Briefly, data were collected using a Siemens 3 T Skyra scanner with a 32-channel head coil. Scan parameters were as follows: TR = 0.72 s, TE = 33.1 ms, flip angle = 52°, field of view = 280 × 180 mm^2^, matrix = 140 × 90, echo spacing = 0.58 ms, bandwidth = 2290 Hz px^−1^. Slice thickness was set to 2.0 mm, 72 slices and 2.0 mm isotropic voxels, multiband acceleration factor = 8. Total scan time was 14.4 min for each resting state scan—one each visit. Each run was completed with oblique axial acquisitions and alternated between phase encoding left-to-right (LR) and phase encoding right-to-left (RL). The open-source HCP minimal preprocessed resting-state fMRI data were used in this work^70^. RIPTiDe was conducted on the cleaned data to quantify and remove the sLFO signal driven by global blood-based physiology^73,74^. Note that negative correlations are an unavoidable consequence of global signal regression, but this is not the case for sLFO regression via RIPTiDe. This is because RIPTiDe adjusts for the time delay of the sLFO signal, allowing for full removal of the sLFO signal specifically and leaving little to no inverted signal in the residuals^75,76^ (e.g., spurious anti-correlations). We confirmed that RIPTiDe significantly reduced the correlation between a brain network dynamics and sLFO dynamics (Fig. S6).

#### Pharmacological manipulation cohort resting-state scan

Data were collected using a Siemens Trio 3 T scanner with a 12-channel RF coil. The 8-min resting state scan was acquired with gradient-echo, echo planar images using oblique axial scans 30° from AC-PC with AP phase encoding and the following parameters (TR = 2 s, TE = 27 ms, flip angle = 78°, voxel resolution = 3.4375 × 3.4375 × 4 mm). Resting-state fMRI data were processed using the FMRIB software library (FSL6.0.0). Images underwent brain extraction, registration, spatial smoothing (6 mm), high-pass temporal filtering (100 s), and motion correction via MCFLIRT. Following motion correction, Independent Component Analysis via FIX was employed using a training set derived from the current data to further clean data^77,78^. Next, RIPTiDe was conducted on the data to quantify and remove the temporal sLFO signal driven by global blood-based physiology. For functional connectivity analysis, TRs with motion greater than 0.35 mm (Euclidean distance) were discarded.

### Deriving the mean sLFO amplitude

We derived the mean brain-wide sLFO amplitude first by running regressor interpolation at progressive time delays (RIPTiDe^73,74^) using the open source “rapidtide” suite (https://github.com/bbfrederick/rapidtidehttps://doi.org/10.5281/zenodo.17633117). RIPTiDe was conducted to derive the mean sLFO amplitude prior to FIX correction, as such correction can interfere with sLFO extraction. RIPTiDe outputs a brain-wide map of squared maximum correlation for each brain voxel, where the max correlation is the correlation between the “probe” signal (global signal) and each voxel’s BOLD signal at the optimal time delay (i.e., at the time delay which allows for maximum correlation). The squared correlation is the fraction of the variance in the voxel explained by the sLFO signal. The mean brain-wide sLFO amplitude is defined as the average of the brain-wide max correlation squared. All subsequent analyses on sLFO × functional brain network relationships *only* use the mean brain-wide sLFO amplitude measure of the sLFO, as this sLFO derivative specifically is the established index of arousal^23^.

### Functional network-network connectivity

To derive network-network functional connectivity values, we extracted mean time courses of each of the seven functional brain networks using the Yeo 7-network parcellation atlas^79^ and FSL (fslmeants) or AFNI (3dMean). We derived the Fisher r-to-z transformed Pearson’s correlation coefficient (*r*) between each pair of network-network time courses (21 correlations per subject). For HCP-YA, r-to-z correlation values were derived for each scan session, and each phase encode direction (four total). For the pharmacological manipulation sample, r-to-z correlation values were derived under each drug condition.

### Mean sLFO × functional network-network connectivity

To test for relationships between the mean sLFO and network-network functional connectivity broadly, we began by conducting 21 linear mixed models, which examined the relationship between sLFO and network-network connectivity while controlling for age, sex, scan session, phase, and within-subject random effects, using the HCP-YA sample.

We then focused on the interactions between the DMN and other functional brain networks, given the importance of DMN-network connectivity in psychopathology, cognition, and demonstrated relationships between DMN-network connectivity and physiological arousal^2,5,8,48,50,54,58^. We conducted a linear mixed model, which tested the interaction between sLFO × DMN-network (DMN-DAN, DMN-FPN, DMN-Limbic, DMN-SN, DMN-SMN, DMN-VIS) in relation to functional connectivity, while controlling for main effects of sLFO, DMN-network, age, sex, scan session, phase encode direction, and within-subject random effects. This was carried out using the mixed function of the afex package in R. After finding a significant sLFO × DMN-network interaction, we conducted post-hoc analysis to compare the strength of sLFO slopes predicting DMN-network functional connectivity across the six DMN-network relationships, resulting in 15 pair-wise tests. This post-hoc analysis was conducted using the emtrends function of the emmeans package in R; significance was corrected for multiple comparisons using the Bonferroni method. Finally, we confirmed that the presence of the temporal sLFO in BOLD data does not drive relationships between mean sLFO amplitude and networks by testing mean sLFO amplitude x DMN-network connectivity relationships before and after RIPTiDe to remove the temporal sLFO data (Fig. S7).

### Reproducibility of mean sLFO × functional network-network connectivity

To confirm the reproducibility of relationships between mean sLFO and DMN-network correlations across separate scan days within the HCP-YA sample, we used Bayes factor analysis. For each DMN-network (DMN-DAN, DMN-Limbic, etc.), we compared a null model to an alternate model. The null modeled the relationship between mean sLFO and network-network connectivity while controlling for session, age, sex, and within-subject random effects. The alternate model solely differed from the null by including a mean sLFO × session interaction. Bayes factors that favor the null (BF > 1) provide evidence for reproducibility, as the sLFO slope would not vary by session. In contrast, favoring the alternate (BF < 1) provides evidence for a session-based difference in the relationship between sLFO and DMN-network connectivity. Similarly, we used Bayes factor analysis to determine reproducibility of sLFO × DMN-network relationships across independent samples: the HCP-YA sample and our sample of 59 healthy adults under placebo (from the pharmacological manipulation trial). In this case, the null modeled the relationship between mean sLFO and DMN-network connectivity while controlling for sample, age, sex, and within-subject random effects. The alternate model solely differed from the null by including an sLFO × sample interaction. Thus, here a Bayes factor that supports the null provides evidence for reproducibility across samples. In contrast, a Bayes factor that supports the alternative provides evidence of a sample-based difference in the relationship between sLFO and network-network connectivity. To conduct Bayes Factor analysis, we used the BayesFactor package in R and used Monte Carlo integration with the number of sampling iterations equal to 100,000. Bayes factors were qualitatively interpreted based on Lee and Wagenmakers^42^, with the following evidence thresholds: anecdotal (1–3), moderate (3–10), strong (10–30), and very strong (30–100). Reciprocal values indicate evidence of equal strength in the alternative direction.

### Time spent in functional network states

To derive the time-varying activity of functional brain networks, we conducted co-activation pattern (CAP) analysis. Specifically, we applied 8 CAPs, which were previously defined by the HCP-YA sample, have been shown to be reliably active at rest, and align with known functional brain networks, including the DMN, DAN, FPN, SN, and SMN^43^. Given the spatial overlap between these data-driven CAPs and functional brain networks, we refer to the CAPs as “functional brain network states.” In conducting CAP analysis on resting-state data, we derived the percentage of time spent in each brain network state at rest as our metric of interest.

### Mean sLFO × time spent in functional network states

To test relationships between the mean sLFO and percent of time in brain network state, we conducted a linear mixed model, which tested for an interaction between sLFO and brain state (FI-DMN, FPN, DMN, DAN, SN-1, SMN, SM-DMN, SN-2) in relation to percentage of time in brain network state, while controlling for age, sex, scan session, phase encode direction, and within-subject random effects. This was carried out using the mixed function of the afex package in R. Finding a significant sLFO ×brain state interaction, we ran a linear model testing the relationship between sLFO and the percentage of time in each brain state. We also calculated sLFO × time in state *R* values.

Finally, given the demonstrated relationship of the SN in coordinating the activity of the DMN and DAN, and our focus on such networks in subsequent analysis, we tested whether time in the SN was directly related to time in the DMN and the DAN via two correlation tests.

### Reproducibility of mean sLFO × time spent in functional network states

To explore the reproducibility of our HCP-YA findings in an independent sample, we followed precisely the same Bayes factor analysis approach as described in the sLFO × DMN-network connectivity analysis. The first set of eight Bayes factors tested for evidence of reproducibility across sessions, within the HCP-YA sample, by comparing the strength of the null model (no sLFO × session interaction) to the alternate (sLFO × session interaction). The second set of eight Bayes factors tested for evidence for reproducibility across independent samples, the HCP-YA sample and the healthy adults placebo cohort, by comparing the strength of the null model (no sLFO × sample interaction) to the alternate (sLFO × sample interaction). Again, in both cases, Bayes factors which provide evidence for the null model support reproducibility (BF > 1), whereas Bayes factors which provide evidence for the alternate provide evidence of a session or sample-based difference in the relationship between sLFO and time spent in functional network states (BF < 1).

### Within-subject changes in mean sLFO and functional brain network properties

In addition to testing relationships between the mean sLFO and functional network properties *between* subjects, we also sought to test whether within-subject changes in mean sLFO were related to within-subject changes in functional brain network properties. We calculated the change in mean sLFO from session 1 to session 2. We also calculated the change in (1) DMN-network connectivity values and (2) percentage of time in brain state from session 1 to session 2. We then ran two linear regression models to test the relationships between (1) ΔsLFO and ΔDMN-network connectivity and (2) ΔsLFO and Δtime in state, which controlled for age, sex, and phase encode direction. Post-hoc analysis included running linear models for each DMN-network/brain state and calculating the *R* values for each correlation across each phase encode direction. Post-hoc analysis for DMN-network connectivity also compared the strength of ΔsLFO slopes across networks, mirroring the between-subjects approach.

### Replicating mean sLFO × brain relationships with traditional arousal indices

We aimed to confirm that the relationships between mean sLFO and (1) DMN-network connectivity and (2) time spent in functional network states mirrored physiology × brain relationships using more traditional indices. The alternate physiological indices include RVT, HF-HRV, and LF-HRV. In this analysis, 29 subjects were missing physiological data, so our total *N* = 433. The models contained one average measurement indexing physiology for each subject and each scan run, mirroring our sLFO-based models. Where a physiology index × network interaction was significant in predicting connectivity, post-hoc analysis was conducted in the same manner as the sLFO analysis.

### Mean sLFO, functional brain networks, and catecholaminergic pharmacology

In our pharmacological manipulation cohort, we tested whether catecholaminergic modulation via pharmacology impacted the relationship between sLFO and (1) DMN-network connectivity and (2) time spent in functional network states through two linear models which tested the effect of sLFO, an sLFO × drug interaction, an sLFO × network interaction, and a three way sLFO × drug × network interaction in relating to connectivity (first linear model) and time spent in network states (second linear model) while controlling for age, sex, and drug. Following significant sLFO × network interactions and no drug × sLFO × network interactions, we ran linear models for each DMN-network/brain state which controlled for age, sex, drug, and within subject random effects.

Next, we tested whether drug-induced changes in sLFO were related to drug-induced changes in DMN-network connectivity and time spent in network states. We tested how changes in sLFO (drug – placebo) relate to changes in (1) DMN-DAN and DMN-SN connectivity and (2) time spent in DMN, DAN, and SN (drug – placebo) through simple correlation tests. This resulted in 10 (5 × 2) correlation tests: sLFO × 5 *y*-variables (DMN-DAN; DMN-SN; DMN_time_; DAN_time_; SN_time_) × 2 drug conditions (methylphenidate – placebo; haloperidol – placebo).

### Reporting summary

Further information on research design is available in the Nature Portfolio Reporting Summary linked to this article.

## Supplementary information

Supplementary Information

Description of Additional Supplementary Files

Supplementary Data 1

Reporting Summary

## Data Availability

Data collected at the National Institute on Drug Abuse (NIDA) requires a data use agreement in order to be shared, which may be established per request by reaching out to the PI. Data from the Human Connectome Project is publicly available on the open-access Connectome database (https://db.humanconnectome.org/app/template/Login.vm). Source data underlying the figures can be found in Supplementary Data 1.

## Code availability

The code and instructions for performing RIPTiDe denoising using the rapidtide package are publicly available at https://rapidtide.readthedocs.io/en/latest/usage_rapidtide.html#removing-low-frequency-physiological-noise-from-fmri-data.

## References

[1] Raut, R. V. et al. Arousal as a universal embedding for spatiotemporal brain dynamics. *Nature* **647**, 454–461 (2025). 10.1038/s41586-025-09544-4 PMC12611781 40993399

[2] Fan, J. et al. Spontaneous brain activity relates to autonomic arousal. *J. Neurosci.* **32**, 11176–11186 (2012). 10.1523/JNEUROSCI.1172-12.2012 PMC3435430 22895703

[3] He, H., Hong, L. & Sajda, P. Pupillary response is associated with the reset and switching of functional brain networks during salience processing. *PLoS Comput. Biol.* **19**, e1011081 (2023). 10.1371/journal.pcbi.1011081 PMC10208478 37172067

[4] Lee, K. et al. Arousal impacts distributed hubs modulating the integration of brain functional connectivity. *NeuroImage* **258**, 119364 (2022). 10.1016/j.neuroimage.2022.119364 PMC9341222 35690257

[5] Liu, T. T. & Falahpour, M. Vigilance effects in resting-state fMRI. *Front. Neurosci.* **14**, 321 (2020). 10.3389/fnins.2020.00321 PMC7190789 32390792

[6] Raut, R. V. et al. Global waves synchronize the brain’s functional systems with fluctuating arousal. *Sci. Adv.* **7**, eabf2709 (2021). 10.1126/sciadv.abf2709 PMC8294763 34290088

[7] Schneider, M. et al. Spontaneous pupil dilations during the resting state are associated with activation of the salience network. *NeuroImage* **139**, 189–201 (2016). 10.1016/j.neuroimage.2016.06.011 27291493

[8] Wong, C. W., Olafsson, V., Tal, O. & Liu, T. T. The amplitude of the resting-state fMRI global signal is related to EEG vigilance measures. *NeuroImage* **83**, 983–990 (2013). 10.1016/j.neuroimage.2013.07.057 PMC3815994 23899724

[9] Young, C. B. et al. Dynamic shifts in large-scale brain network balance as a function of arousal. *J. Neurosci.* **37**, 281–290 (2017). 10.1523/JNEUROSCI.1759-16.2016 PMC6596574 28077708

[10] Chang, C. et al. Tracking brain arousal fluctuations with fMRI. *Proc. Natl. Acad. Sci. USA* **113**, 4518–4523 (2016). 10.1073/pnas.1520613113 PMC4843437 27051064

[11] Liu, X. et al. Subcortical evidence for a contribution of arousal to fMRI studies of brain activity. *Nat. Commun.* **9**, 395 (2018). 10.1038/s41467-017-02815-3 PMC5786066 29374172

[12] van Es, M. W. J. et al. Large-scale cortical functional networks are organized in structured cycles. *Nat. Neurosci.* **28**, 2118–2128 (2025). 10.1038/s41593-025-02052-8 PMC12497652 40866607

[13] Wang, C., Ong, J. L., Patanaik, A., Zhou, J. & Chee, M. W. L. Spontaneous eyelid closures link vigilance fluctuation with fMRI dynamic connectivity states. *Proc. Natl. Acad. Sci. USA* **113**, 9653–9658 (2016). 10.1073/pnas.1523980113 PMC5003283 27512040

[14] Xia, C. H. et al. Linked dimensions of psychopathology and connectivity in functional brain networks. *Nat. Commun.* **9**, 3003 (2018). 10.1038/s41467-018-05317-y PMC6070480 30068943

[15] Yellin, D., Berkovich-Ohana, A. & Malach, R. Coupling between pupil fluctuations and resting-state fMRI uncovers a slow build-up of antagonistic responses in the human cortex. *NeuroImage* **106**, 414–427 (2015). 10.1016/j.neuroimage.2014.11.034 25463449

[16] Aston-Jones, G. & Cohen, J. D. An integrative theory of locus coeruleus-norepinephrine function: adaptive gain and optimal performance. *Annu. Rev. Neurosci.* **28**, 403–450 (2005). 10.1146/annurev.neuro.28.061604.135709 16022602

[17] Davidson, E. J. & Spreng, R. N. Anticorrelation emerges within a dynamic and competitive neural landscape. *Curr. Opin. Behav. Sci.* **67**, 101621 (2026).

[18] Satpute, A. B., Kragel, P. A., Barrett, L. F., Wager, T. D. & Bianciardi, M. Deconstructing arousal into wakeful, autonomic and affective varieties. *Neurosci. Lett.* **693**, 19–28 (2019). 10.1016/j.neulet.2018.01.042 PMC6068010 29378297

[19] Petersen, S. E. & Posner, M. I. The attention system of the human brain: 20 years after. *Annu. Rev. Neurosci.* **35**, 73–89 (2012). 10.1146/annurev-neuro-062111-150525 PMC3413263 22524787

[20] Tong, Y., Hocke, L. M. & Frederick, B. B. Low frequency systemic hemodynamic “noise” in resting state BOLD fMRI: characteristics, causes, implications, mitigation strategies, and applications. *Front. Neurosci.* **13**, 787 (2019). 10.3389/fnins.2019.00787 PMC6702789 31474815

[21] Tong, Y. et al. Evaluating the effects of systemic low frequency oscillations measured in the periphery on the independent component analysis results of resting state networks. *NeuroImage* **76**, 202–215 (2013). 10.1016/j.neuroimage.2013.03.019 PMC3652630 23523805

[22] Bolt, T. et al. Autonomic physiological coupling of the global fMRI signal. *Nat. Neurosci.* **28**, 1327–1335 (2025). 10.1038/s41593-025-01945-y PMC13725885 40335772

[23] Korponay, C., Janes, A. C. & Frederick, B. B. Brain-wide functional connectivity artifactually inflates throughout functional magnetic resonance imaging scans. *Nat. Hum. Behav.* **8**, 1568–1580 (2024). 10.1038/s41562-024-01908-6 PMC11526723 38898230

[24] Kaiser, R. H. et al. Dynamic resting-state functional connectivity in major depression. *Neuropsychopharmacology* **41**, 1822–1830 (2016). 10.1038/npp.2015.352 PMC4869051 26632990

[25] Kaiser, R. H. et al. Abnormal frontoinsular-default network dynamics in adolescent depression and rumination: a preliminary resting-state co-activation pattern analysis. *Neuropsychopharmacology* **44**, 1604–1612 (2019). 10.1038/s41386-019-0399-3 PMC6784913 31035283

[26] Quam, A., Biernacki, K., Ross, T. J., Salmeron, B. J. & Janes, A. C. Childhood trauma, emotional awareness, and neural correlates of long-term nicotine smoking. *JAMA Netw. Open* **7**, e2351132 (2024). 10.1001/jamanetworkopen.2023.51132 PMC10784870 38206627

[27] Wang, K. S. et al. Nicotine acutely alters temporal properties of resting brain states. *Drug Alcohol Depend.* **226**, 108846 (2021). 10.1016/j.drugalcdep.2021.108846 PMC8355138 34198131

[28] Gu, H., Schulz, K. P., Fan, J. & Yang, Y. Temporal dynamics of functional brain states underlie cognitive performance. *Cereb. Cortex* **31**, 2125–2138 (2021). 10.1093/cercor/bhaa350 PMC7945029 33258911

[29] Biernacki, K. et al. From rest to focus: pharmacological modulation of the relationship between resting state dorsal attention network dynamics and task-based brain activation. *Neuropsychopharmacology* **51**, 926–933 (2026). 10.1038/s41386-025-02318-6 PMC13013587 41530553

[30] Peterson, E. C., Smolker, H. R., Moser, A. D. & Kaiser, R. H. Review of dynamic resting-state methods in neuroimaging: applications to depression and rumination. *Hum. Brain Mapp.* **46**, e70377 (2025). 10.1002/hbm.70377 PMC12532080 41104784

[31] Preti, M. G., Bolton, T. A. & Van De Ville, D. The dynamic functional connectome: state-of-the-art and perspectives. *NeuroImage* **160**, 41–54 (2017). 10.1016/j.neuroimage.2016.12.061 28034766

[32] Chang, X. et al. Neurocognitive characterization of behaviour and mental illness through time-varying brain network analysis. *Nat. Commun.* **17**, 1353 (2026). 10.1038/s41467-025-67398-w PMC12877127 41633990

[33] España, R. A., Schmeichel, B. E. & Berridge, C. W. Norepinephrine at the nexus of arousal, motivation and relapse. *Brain Res.* **1641**, 207–216 (2016). 10.1016/j.brainres.2016.01.002 PMC4879075 26773688

[34] Kaźmierczak, M. & Nicola, S. M. The arousal-motor hypothesis of dopamine function: evidence that dopamine facilitates reward seeking in part by maintaining arousal. *Neuroscience* **499**, 64–103 (2022). 10.1016/j.neuroscience.2022.07.008 PMC9479757 35853563

[35] Ross, J. A. & Van Bockstaele, E. J. The role of catecholamines in modulating responses to stress: sex-specific patterns, implications, and therapeutic potential for post-traumatic stress disorder and opiate withdrawal. *Eur. J. Neurosci.* **52**, 2429–2465 (2020). 10.1111/ejn.14714 PMC8351794 32125035

[36] Sara, S. J. & Bouret, S. Orienting and reorienting: the locus coeruleus mediates cognition through arousal. *Neuron* **76**, 130–141 (2012). 10.1016/j.neuron.2012.09.011 23040811

[37] Ranjbar-Slamloo, Y. & Fazlali, Z. Dopamine and noradrenaline in the brain; overlapping or dissociate functions?. *Front. Mol. Neurosci.* **12**, 334 (2020). 10.3389/fnmol.2019.00334 PMC6986277 32038164

[38] Hill, J. A., Korponay, C., Salmeron, B. J., Ross, T. J. & Janes, A. C. Catecholaminergic modulation of large-scale network dynamics is tied to the reconfiguration of corticostriatal connectivity. *Hum. Brain Mapp.* **45**, e70086 (2024). 10.1002/hbm.70086 PMC11635694 39665506

[39] Rosenberg, M. D. et al. Methylphenidate modulates functional network connectivity to enhance attention. *J. Neurosci.* **36**, 9547–9557 (2016). 10.1523/JNEUROSCI.1746-16.2016 PMC5039242 27629707

[40] Pedersen, R. et al. Dopamine D1-receptor organization contributes to functional brain architecture. *J. Neurosci.* **44**, e0621232024 (2024). 10.1523/JNEUROSCI.0621-23.2024 PMC10941071 38302439

[41] Tong, C. et al. Norepinephrine-mediated arousal fluctuations drive inverted U-shaped functional connectivity dynamics. *Nat. Commun.* **16**, 11318 (2025). 10.1038/s41467-025-66436-x PMC12722237 41390822

[42] Martin, C. G., He, B. J. & Chang, C. State-related neural influences on fMRI connectivity estimation. *Neuroimage* **244**, 118590 (2021). 10.1016/j.neuroimage.2021.118590 PMC8815005 34560268

[43] Lee, M. & Wagenmakers, E.-J. *Bayesian Cognitive Modeling: A Practical Course* (Cambridge University Press, 2014).

[44] Janes, A. C., Peechatka, A. L., Frederick, B. B. & Kaiser, R. H. Dynamic functioning of transient resting-state coactivation networks in the Human Connectome Project. *Hum. Brain Mapp.* **41**, 373–387 (2020). 10.1002/hbm.24808 PMC7268046 31639271

[45] Dang, L. C., O’Neil, J. P. & Jagust, W. J. Dopamine supports coupling of attention-related networks. *J. Neurosci.* **32**, 9582–9587 (2012). 10.1523/JNEUROSCI.0909-12.2012 PMC3410707 22787044

[46] Gao, Y. et al. Decreased dorsal attention network homogeneity as a potential neuroimaging biomarker for major depressive disorder. *J. Affect. Disord.* **332**, 136–142 (2023). 10.1016/j.jad.2023.03.080 36990286

[47] Huang, H. et al. Resting-state functional connectivity of salience network in schizophrenia and depression. *Sci. Rep.* **12**, 11204 (2022). 10.1038/s41598-022-15489-9 PMC9249853 35778603

[48] McCutcheon, R. A. et al. Mesolimbic dopamine function is related to salience network connectivity: an integrative positron emission tomography and magnetic resonance study. *Biol. Psychiatry* **85**, 368–378 (2019). 10.1016/j.biopsych.2018.09.010 PMC6360933 30389131

[49] Owens, M. M. et al. Investigation of psychiatric and neuropsychological correlates of default mode network and dorsal attention network anticorrelation in children. *Cereb. Cortex* **30**, 6083–6096 (2020). 10.1093/cercor/bhaa143 PMC8086768 32591777

[50] Schimmelpfennig, J., Topczewski, J., Zajkowski, W. & Jankowiak-Siuda, K. The role of the salience network in cognitive and affective deficits. *Front. Hum. Neurosci.* **17**, 1133367 (2023). 10.3389/fnhum.2023.1133367 PMC10067884 37020493

[51] Zhang, R. & Volkow, N. D. Brain default-mode network dysfunction in addiction. *Neuroimage* **200**, 313–331 (2019). 10.1016/j.neuroimage.2019.06.036 31229660

[52] Mather, M., Clewett, D., Sakaki, M. & Harley, C. W. Norepinephrine ignites local hotspots of neuronal excitation: How arousal amplifies selectivity in perception and memory. *Behav. Brain Sci.* **39**, e200 (2016). 10.1017/S0140525X15000667 PMC5830137 26126507

[53] Anticevic, A. et al. The role of default network deactivation in cognition and disease. *Trends Cogn. Sci.* **16**, 584–592 (2012). 10.1016/j.tics.2012.10.008 PMC3501603 23142417

[54] Kucyi, A. et al. Electrophysiological dynamics of antagonistic brain networks reflect attentional fluctuations. *Nat. Commun.* **11**, 325 (2020). 10.1038/s41467-019-14166-2 PMC6965628 31949140

[55] Menon, V. Large-scale brain networks and psychopathology: a unifying triple network model. *Trends Cogn. Sci.* **15**, 483–506 (2011). 10.1016/j.tics.2011.08.003 21908230

[56] Haider, B., Häusser, M. & Carandini, M. Inhibition dominates sensory responses in awake cortex. *Nature* **493**, 97–100 (2013). 10.1038/nature11665 PMC3537822 23172139

[57] Foote, S. L., Freedman, R. & Oliver, A. P. Effects of putative neurotransmitters on neuronal activity in monkey auditory cortex. *Brain Res.* **86**, 229–242 (1975). 10.1016/0006-8993(75)90699-x 234774

[58] Polack, P.-O., Friedman, J. & Golshani, P. Cellular mechanisms of brain-state-dependent gain modulation in visual cortex. *Nat. Neurosci.* **16**, 1331–1339 (2013). 10.1038/nn.3464 PMC3786578 23872595

[59] Menon, V. 20 years of the default mode network: a review and synthesis. *Neuron* **111**, 2469–2487 (2023). 10.1016/j.neuron.2023.04.023 PMC10524518 37167968

[60] Spreng, R. N., Stevens, W. D., Chamberlain, J. P., Gilmore, A. W. & Schacter, D. L. Default network activity, coupled with the frontoparietal control network, supports goal-directed cognition. *NeuroImage* **53**, 303–317 (2010). 10.1016/j.neuroimage.2010.06.016 PMC2914129 20600998

[61] Markovic, J., Anderson, A. K. & Todd, R. M. Tuning to the significant: Neural and genetic processes underlying affective enhancement of visual perception and memory. *Behav. Brain Res.* **259**, 229–241 (2014). 10.1016/j.bbr.2013.11.018 24269973

[62] Menon, V. & Uddin, L. Q. Saliency, switching, attention and control: a network model of insula function. *Brain Struct. Funct.* **214**, 655–667 (2010). 10.1007/s00429-010-0262-0 PMC2899886 20512370

[63] Sridharan, D., Levitin, D. J. & Menon, V. A critical role for the right fronto-insular cortex in switching between central-executive and default-mode networks. *Proc. Natl. Acad. Sci. USA* **105**, 12569–12574 (2008). 10.1073/pnas.0800005105 PMC2527952 18723676

[64] Weber, S., Aleman, A. & Hugdahl, K. Involvement of the default mode network under varying levels of cognitive effort. *Sci. Rep.* **12**, 6303 (2022). 10.1038/s41598-022-10289-7 PMC9012747 35428802

[65] Chen, Z., Li, G. & Liu, J. Autonomic dysfunction in Parkinson’s disease: Implications for pathophysiology, diagnosis, and treatment. *Neurobiol. Dis.* **134**, 104700 (2020). 10.1016/j.nbd.2019.104700 31809788

[66] Doolittle, J. D. Could treatment of autonomic dysfunction in early and prodromal Parkinson’s disease slow disease progression?. *J. Neurophysiol.* **129**, 1279–1281 (2023). 10.1152/jn.00380.2022 37141072

[67] Sgoifo, A., Carnevali, L., Pico Alfonso, M. D. L. A. & Amore, M. Autonomic dysfunction and heart rate variability in depression. *Stress* **18**, 343–352 (2015). 10.3109/10253890.2015.1045868 26004818

[68] Alvares, G. A., Quintana, D. S., Hickie, I. B. & Guastella, A. J. Autonomic nervous system dysfunction in psychiatric disorders and the impact of psychotropic medications: a systematic review and meta-analysis. *J. Psychiatry Neurosci.* **41**, 89–104 (2016). 10.1503/jpn.140217 PMC4764485 26447819

[69] Isaac, V., Lopez, V. & Escobar, M. J. Arousal dysregulation and executive dysfunction in attention deficit hyperactivity disorder (ADHD). *Front. Psychiatry* **14**, 1336040 (2024). 10.3389/fpsyt.2023.1336040 PMC10827919 38298926

[70] Fu, Q. Autonomic dysfunction and cardiovascular risk in post-traumatic stress disorder. *Auton. Neurosci.* **237**, 102923 (2022). 10.1016/j.autneu.2021.102923 34844132

[71] Welsh, J. C. et al. Functional magnetic resonance imaging signal typically viewed as “noise” has clinical relevance in psychiatry. *Biol. Psychiatry Glob. Open Sci.* **6**, 100720 (2026). 10.1016/j.bpsgos.2026.100720 PMC13090997 42005285

[72] Glasser, M. F. et al. The Human Connectome Project’s neuroimaging approach. *Nat. Neurosci.* **19**, 1175–1187 (2016). 10.1038/nn.4361 PMC6172654 27571196

[73] Van Essen, D. C. et al. The WU-Minn Human Connectome Project: an overview. *Neuroimage* **80**, 62–79 (2013). 10.1016/j.neuroimage.2013.05.041 PMC3724347 23684880

[74] Frederick, B. D., Nickerson, L. D. & Tong, Y. Physiological denoising of BOLD fMRI data using Regressor Interpolation at Progressive Time Delays (RIPTiDe) processing of concurrent fMRI and near-infrared spectroscopy (NIRS). *Neuroimage* **60**, 1913–1923 (2012). 10.1016/j.neuroimage.2012.01.140 PMC3593078 22342801

[75] Tong, Y. & Frederick, B. D. Time lag dependent multimodal processing of concurrent fMRI and near-infrared spectroscopy (NIRS) data suggests a global circulatory origin for low-frequency oscillation signals in human brain. *Neuroimage* **53**, 553–564 (2010). 10.1016/j.neuroimage.2010.06.049 PMC3133965 20600975

[76] Erdoğan, S. B., Tong, Y., Hocke, L. M., Lindsey, K. P. & deB Frederick, B. Correcting for blood arrival time in global mean regression enhances functional connectivity analysis of resting state fMRI-BOLD signals. *Front. Hum. Neurosci.* **10**, 311 (2016). 10.3389/fnhum.2016.00311 PMC4923135 27445751

[77] Griffanti, L. et al. ICA-based artefact removal and accelerated fMRI acquisition for improved resting state network imaging. *Neuroimage* **95**, 232–247 (2014). 10.1016/j.neuroimage.2014.03.034 PMC4154346 24657355

[78] Salimi-Khorshidi, G. et al. Automatic denoising of functional MRI data: combining independent component analysis and hierarchical fusion of classifiers. *Neuroimage* **90**, 449–468 (2014). 10.1016/j.neuroimage.2013.11.046 PMC4019210 24389422

[79] Thomas Yeo, B. T. et al. The organization of the human cerebral cortex estimated by intrinsic functional connectivity. *J. Neurophysiol.* **106**, 1125–1165 (2011). 10.1152/jn.00338.2011 PMC3174820 21653723

