# Supplementary material for: Physiological arousal as a predominant source of individual differences in functional brain networks
Source: Commun Biol. 2026 Jul 10;9:1446. doi: 10.1038/s42003-026-10624-y (PMC13728107; doi:10.1038/s42003-026-10624-y)
Supplement: Supplementary file 1 [file 42003_2026_10624_MOESM1_ESM.pdf]

Supplementary Results

Figure S1.

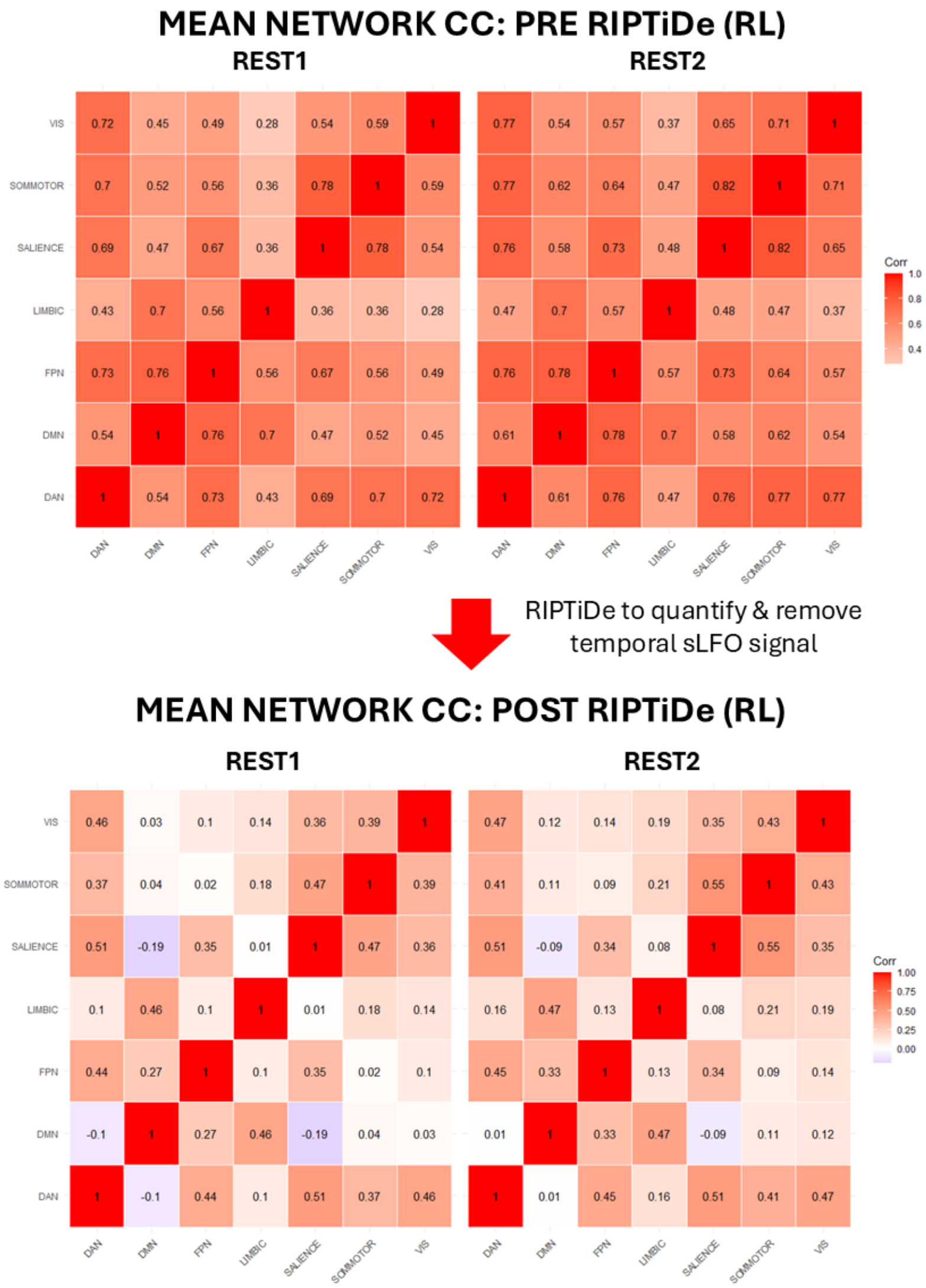

Figure S1. Mean network-network connectivity values across all 7 functional networks before and after RIPTiDe. After RIPTiDe, there is less positive global mean functional connectivity, confirming that regression of sLFO signal using RIPTiDe package reduced global mean functional connectivity. Data based on n = 462 from the Human Connectome Project.

Table S1.

**Network-network**	**F**	**Den df**	**p (corrected)**
**DAN-FPN**	108.45	1830.94	p<0.001
**DAN-LIM**	1201.16	1805.16	<0.001
**DAN-SMO**	1118.79	1795.08	<0.001
**DAN-SN**	155.82	1776.90	<0.001
**DAN-VIS**	1131.31	1765.98	<0.001
**FPN-LIM**	296.34	1801.24	<0.001
**FPN-SMO**	632.55	1779.585	<0.001
**FPN-SN**	735.40	1832.79	<0.001
**FPN-VIS**	551.96	1772.865	<0.001
**LIM-SMO**	971.065	1689.14	<0.001
**LIM-SN**	736.015	1789.32	<0.001
**LIM-VIS**	1043.64	1709.30	<0.001
**SMO-VIS**	825.12	1832.03	<0.001
**SN-SMO**	447.035	1817.92	<0.001
**SN-VIS**	199.98	1793.41	<0.001

Table S1. Results for sLFO term in models testing relationship between sLFO and network-network connectivity while controlling for age, sex, session, and phase, for all network-network relationships *except* DMN-network relationships (see Table S2). Data based on n = 462 from the Human Connectome Project.

Table S2.

Model Term	num Df	den Df	F	Pr(>F)	NETWORK
sLFO	1	1817.09	1667.98	<0.001	DMN-DAN
age	1	462.03	2.66	0.10	DMN-DAN
sex	1	463.17	0.30	0.58	DMN-DAN
session	1	1383.61	2.55	0.11	DMN-DAN
phase	1	1383.49	1.11	0.29	DMN-DAN
sLFO	1	1832.10	600.15	<0.001	DMN-FPN
age	1	460.88	1.47	0.23	DMN-FPN
sex	1	461.975	0.83	0.36	DMN-FPN
session	1	1382.54	0.35	0.55	DMN-FPN
phase	1	1382.43	0.46	0.50	DMN-FPN
sLFO	1	1727.78	206.73	<0.001	DMN-LIMBIC
age	1	460.03	8.63	0.0035	DMN-LIMBIC
sex	1	461.28	0.92	0.34	DMN-LIMBIC
session	1	1381.45	0.024	0.88	DMN-LIMBIC
phase	1	1381.32	1.82	0.18	DMN-LIMBIC
sLFO	1	1828.16	1360.12	<0.001	DMN-SN
age	1	461.73	0.52	0.47	DMN-SN
sex	1	462.84	9.72	0.0019	DMN-SN
session	1	1383.37	1.09	0.30	DMN-SN
phase	1	1383.25	1.765	0.18	DMN-SN
sLFO	1	1816.14	1286.27	<0.001	DMN-SMN
age	1	462.07	0.62	0.43	DMN-SMN
sex	1	463.22	3.79	0.052	DMN-SMN
session	1	1383.655	2.09	0.15	DMN-SMN
phase	1	1383.53	0.31	0.58	DMN-SMN
sLFO	1	1793.46	1351.82	<0.001	DMN-VIS
age	1	461.85	2.65	0.10	DMN-VIS
sex	1	463.035	7.06	0.0082	DMN-VIS
session	1	1383.37	6.53	0.011	DMN-VIS
phase	1	1383.243	1.49	0.22	DMN-VIS

Table S2. Each DMN-Network model testing relationship between sLFO and network-network connectivity while controlling for age, sex, session, and phase. Data based on n = 462 from the Human Connectome Project.

Table S3.

DMN-Network Contrast	estimate	z.ratio	p
DMN-DAN — DMN-FPN	0.71	14.39	<0.001
DMN-DAN — DMN-LIMBIC	1.14	23.16	<0.001
DMN-DAN — DMN-SALIENCE	-0.02	-0.41	0.998452
DMN-DAN — DMN-SOMMOTOR	0.28	5.67	<0.001
DMN-DAN — DMN-VIS	0.34	6.82	<0.001
DMN-FPN — DMN-LIMBIC	0.43	8.77	<0.001
DMN-FPN — DMN-SALIENCE	-0.73	-14.80	<0.001
DMN-FPN — DMN-SOMMOTOR	-0.43	-8.72	<0.001
DMN-FPN — VIS	-0.37	-7.57	<0.001
DMN-LIMBIC — DMN-SALIENCE	-1.16	-23.57	<0.001
DMN-LIMBIC — DMN-SOMMOTOR	-0.86	-17.49	<0.001
DMN-LIMBIC — DMN-VIS	-0.805	-16.34	<0.001
DMN-SALIENCE — DMN-SOMMOTOR	0.300	6.09	<0.001
DMN-SALIENCE — DMN-VIS	0.36	7.24	<0.001
DMN-SOMMOTOR — DMN-VIS	0.057	1.15	0.860397

Table S3. Pairwise comparisons between the sLFO slopes for each DMN-network relationship collapsed across session 1 and session 2. Estimate reflects the difference in sLFO slope between the two DMN-network connectivity models. Models controlled for age, sex, phase-encoding direction and session. SE=0.049 and is consistent for each comparison because it is calculated as a result of the whole model which includes each network-network value. Degrees of freedom are asymptotic (approach infinity). Data based on n = 462 from the Human Connectome Project.

Table S4.

Model Term	num Df	den Df	F	p	state
sLFO	1	1769.93	697.40	<0.001	FI-DMN
age	1	461.63	27.10	<0.001	FI-DMN
sex	1	462.84	25.12	<0.001	FI-DMN
phase	1	922.80	0.63	0.43	FI-DMN
session	1	460.67	0.10	0.755	FI-DMN
sLFO	1	1712.70	7.28	0.007	FPN
age	1	462.04	2.52	0.11	FPN
sex	1	463.30	2.49	0.115	FPN
phase	1	921.90	0.06	0.81	FPN
session	1	460.87	38.35	<0.001	FPN
sLFO	1	1786.16	1016.13	<0.001	DMN
age	1	461.70	0.63	0.43	DMN
sex	1	462.88	4.01	0.046	DMN
phase	1	922.83	0.56	0.45	DMN
session	1	460.75	2.22	0.14	DMN
sLFO	1	1829.68	546.99	<0.001	DAN
age	1	461.08	7.75	0.0056	DAN
sex	1	462.18	5.04	0.025	DAN
phase	1	920.92	1.78	0.18	DAN
session	1	460.86	16.10	<0.001	DAN
sLFO	1	1617.47	626.55	<0.001	SN-1
age	1	462.01	4.66	0.031	SN-1
sex	1	463.31	0.14	0.71	SN-1
phase	1	923.06	0.00	0.98	SN-1
session	1	462.02	11.27	0.00085	SN-1
sLFO	1	1698.49	824.27	<0.001	SMO
age	1	462.38	34.79	<0.001	SMO
sex	1	463.65	20.85	<0.001	SMO
phase	1	922.57	1.59	0.21	SMO
session	1	460.64	0.06	0.81	SMO
sLFO	1	1752.31	7.61	0.00585	SM-DMN
age	1	461.27	10.07	0.0016	SM-DMN
sex	1	462.49	11.01	<0.001	SM-DMN
phase	1	922.49	0.73	0.39	SM-DMN
session	1	460.90	2.48	0.12	SM-DMN
sLFO	1	1786.22	0.11	0.74	SN-2
age	1	460.40	47.49	<0.001	SN-2
sex	1	461.58	22.82	<0.001	SN-2

phase	1	922.53	0.00	0.97	SN-2
session	1	460.44	25.39	<0.001	SN-2

Table S4. Each brain state model testing relationship between sLFO and network temporal dynamics while controlling for age, sex, session, and phase. Data based on n = 462 from the Human Connectome Project.

Investigating age-related effects on the relationship between sLFO and brain state temporal dynamics

There was a significant age x sLFO x brain state interaction in the HCP-YA sample (n = 462), in a model predicting percentage of time in state ($F=4.06$, $DOF=7/14121.23$, $p<0.001$). After correcting for eight multiple comparisons, only interactions with SN-1 ($F=8.47$, $DOF=1/1617.69$, $p_{corr}=0.02$) and DAN ($F=7.335$, $DOF=1/1829.47$, $p_{corr}=0.054$) survived, with DAN at trend-level. For both DAN and SN-1, there was a stronger relationship between sLFO and percent of time in brain network state in younger individuals compared to older. Before multiple comparisons corrections, there were also trend-level sLFO x age interactions for the FI-DMN ($F=3.2$, $DOF=1/1766.95$, $p_{raw}=0.07$) and FPN ($F=3.2$, $DOF=1/1718.37$, $p_{raw}=0.07$) states.

Figure S2.

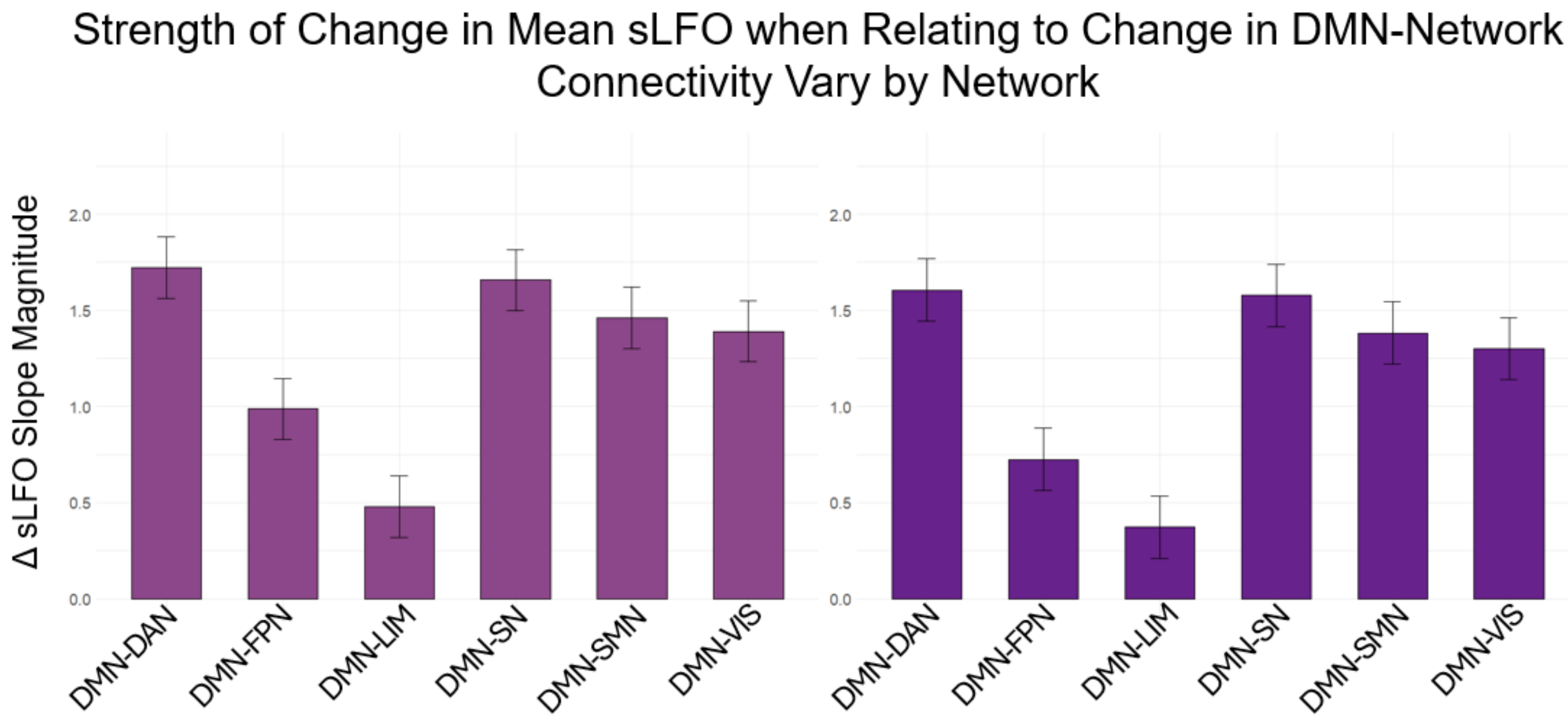

Figure S2. Magnitude of change in sLFO slopes (REST1 – REST2) predicting change in network-network connectivity (REST1-REST2). Each bar represents a single estimated sLFO slope derived from the combined data of all subjects instead of means. The error bars indicate the corresponding confidence intervals around that estimate. First 6 network-network values are data from LR phase encoding. Second 6 network-network values are data from RL phase encoding. Error bars represent upper/lower limits of trend prediction. Data based on n = 462 from the Human Connectome Project.

Table S5.

**DMN-Network Contrast**	**estimate**	**z.ratio**	**p.value**
DMN-DAN – DMN-FPN	0.83	12.175	<0.001
DMN-DAN – LIMBIC	1.33	19.52	<0.001
DMN-DAN – DMN-SALIENCE	0.05	0.75	0.98
DMN-DAN – DMN-SOMMOTOR	0.31	4.60	<0.001
DMN-DAN – DMN-VIS	0.34	4.95	<0.001
DMN-FPN – DMN-LIMBIC	0.50	7.34	<0.001
DMN-FPN – DMN-SALIENCE	-0.78	-11.43	<0.001
DMN-FPN – DMN-SOMMOTOR	-0.52	-7.57	<0.001
DMN-FPN – DMN-VIS	-0.49	-7.23	<0.001
DMN-LIMBIC – DMN-SALIENCE	-1.28	-18.77	<0.001
DMN-LIMBIC – DMN-SOMMOTOR	-1.02	-14.92	<0.001
DMN-LIMBIC – DMN-VIS	-0.99	-14.57	<0.001
DMN-SALIENCE – DMN-SOMMOTOR	0.26	3.85	0.0016
DMN-SALIENCE – DMN-VIS	0.29	4.20	<0.001
DMN-SOMMOTOR – DMN-VIS	0.0235	0.345	0.999

Table S5. Pairwise comparisons between the change in sLFO slope (REST1-REST2) and change in each DMN-network relationship (REST1-REST2). Models controlled for age, sex, and phase encoding. SE=0.07 and is consistent for each comparison because it is calculated as a result of the whole model which includes each network-network value. Degrees of freedom are asymptotic (approach infinity). Data based on n = 462 from the Human Connectome Project.

Table S6.

Model Term	den Df	F	p	state
sLFO (REST2 - REST1)	1843.00	535.73	<0.001	FI-DMN
age	459.00	0.16	0.69	FI-DMN
sex	458.92	0.80	0.37	FI-DMN
phase	1470.29	44.33	<0.001	FI-DMN
sLFO (REST2 - REST1)	1832.56	1.04	0.31	FPN
age	457.96	0.01	0.93	FPN
sex	457.87	2.16	0.14	FPN
phase	1477.08	0.00	0.99	FPN
sLFO (REST2 - REST1)	1833.34	880.56	<0.001	DMN
age	458.52	0.03	0.87	DMN
sex	458.43	0.14	0.71	DMN
phase	1477.29	63.40	<0.001	DMN
sLFO (REST2 - REST1)	1842.06	560.60	<0.001	DAN
age	458.52	2.18	0.14	DAN
sex	458.44	0.03	0.86	DAN
phase	1472.37	50.59	<0.001	DAN
sLFO (REST2 - REST1)	1804.79	547.83	<0.001	SN-1
age	458.02	1.45	0.23	SN-1
sex	457.93	2.01	0.16	SN-1
phase	1483.31	24.08	<0.001	SN-1
sLFO (REST2 - REST1)	1841.81	733.04	<0.001	SMN
age	458.29	0.36	0.55	SMN
sex	458.21	3.56	0.06	SMN
phase	1467.09	72.34	<0.001	SMN
sLFO (REST2 - REST1)	1824.77	9.14	0.0025	SM-DMN
age	458.64	0.01	0.92	SM-DMN
sex	458.55	0.13	0.72	SM-DMN
phase	1479.92	27.49	<0.001	SM-DMN
sLFO (REST2 - REST1)	1842.97	1.87	0.17	SN-2
age	459.01	0.04	0.84	SN-2
sex	458.93	3.97	0.047	SN-2
phase	1470.03	40.43	<0.001	SN-2

Table S6. Within subject F, DOF and p-value for models predicting change in percent time in state from change in sLFO from REST1 to REST2. Num df = 1 for all models/terms. Models controlled for sex, age, and phase. Data based on n = 462 from the Human Connectome Project.

Figure S3.

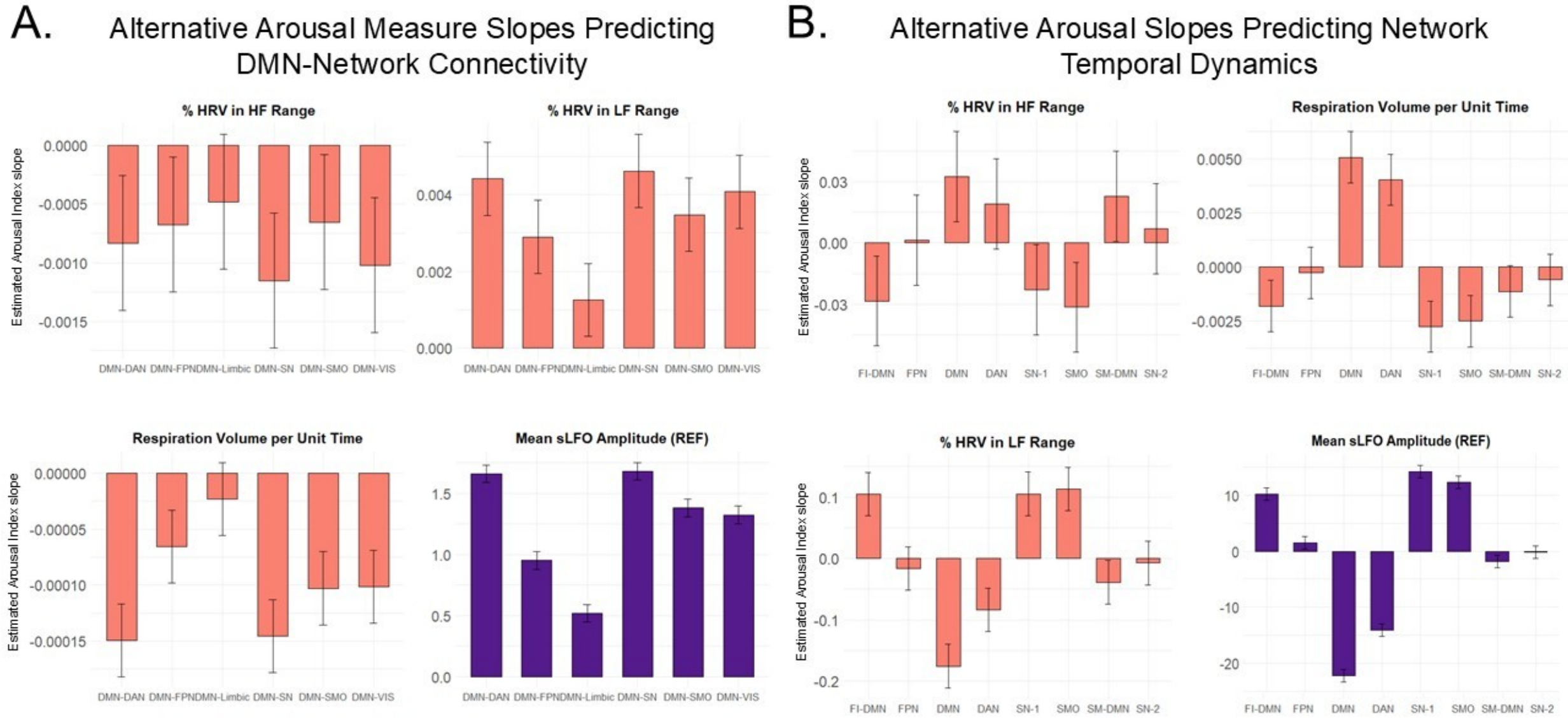

Figure S3. A. Alternative arousal measure slopes predicting DMN-network connectivity by network (n = 462). B. Alternative arousal slopes predicting network temporal dynamics, by brain state. For both plots, orange indicates alternate arousal measure; purple indicates sLFO slope for reference. Each bar represents a single estimated sLFO slope derived from the combined data of all subjects instead of means. The error bars indicate the corresponding confidence intervals around that estimate. Alternative arousal measures include HF-HRV, LF-HRV, and RVT. RVT: Respiration Volume per Unit Time; HF-HRV: Percent of heart rate variability in high frequency range; LF-HRV: Percent of heart rate variability in low frequency range.

We also tested whether alternate indices of arousal predict within subject change in network properties. For changes in static connectivity, we tested whether change in 1) RVT, 2) LF-HRV, 3) HF-HRV from session 1 to session 2 explains changes in DMN-network connectivity from session 1 to session 2, in three independent models which controlled for scan phase encoding, included a network interaction term, and demeaned Δ physiological variables. We found that ΔRVT explains changes in DMN-network connectivity ($F(1, 3968.18)=10.41$, $p=0.001$) and there was a significant network x ΔRVT interaction ($F(5, 4500.60)=6.31$ $p<0.001$) indicating a network-specific effect on the amount that ΔRVT explains ΔDMN-network connectivity. We also found that ΔLF-HRV explains changes in DMN-network connectivity ($F(1, 3171.81)=58.41$, $p<0.001$) and again, there was a significant network x ΔLF-HRV interaction ($F(5, 4500.12)=6.10$ $p<0.001$). Thirdly, we found that ΔHF-HRV did not explain changes in DMN-network connectivity between sessions ($p>0.05$). For the changes in dynamic connectivity, we found that there was a significant interaction between ΔRVT, ΔLF-HRV, ΔHR-HRV and brain state in predicting Δtime in state from session 1 to session 2 (for ΔRVT x brainstate, $F(7, 6607)=12.21$, $p<0.001$; for ΔLF-HRV x brainstate, $F(7, 6607)=23.10$, $p<0.001$; for ΔHL-HRV x brainstate, $F(7, 6607)=4.82$, $p<0.001$).

Table S7

Model term	num Df	den Df	F	p
RVT	1	7915.26	136.24	<0.001
network	5	2145.11	2134.62	<0.001
session	1	7561.98	2.31	0.128598
phase	1	7534.77	0.38	0.536845
sex	1	426.70	10.58	0.0012
age	1	427.30	3.67	0.056
network x RVT	5	7360.72	9.40	<0.001
HF-HRV	1	7513.89	23.31	<0.001
network	5	2144.49	2109.73	<0.001
session	1	7569.11	1.16	0.28
phase	1	7534.47	0.06	0.80
sex	1	438.65	6.84	0.0092
age	1	426.32	1.77	0.185
network x HF-HRV	5	4990.86	0.90	0.48
LF-HRV	1	7840.69	182.56	<0.001
network	5	2144.53	2117.68	<0.001
session	1	7560.41	1.32	0.25
phase	1	7533.74	0.00	0.97
sex	1	430.91	5.02	0.0255
age	1	426.35	1.54	0.215
network x LF-HRV	5	6352.69	7.60	<0.001

Table S7. Three model results for each alternate indices of arousal relating to DMN-network connectivity. All physiology measures (RVT, HF-HRV, LF-HRV) were demeaned in each model. RVT: respiration volume per unit time; HF-HRV: Percent of heart rate variability in high frequency range; LF-HRV: Percent of heart rate variability in low frequency range. Data based on n = 462 from the Human Connectome Project.

Table S8.

INDEX	contrast	estimate	z.ratio	p.value	pcor
	DAN - FPN	-8.39E-05	-3.77	0.0022	0.033462
	DAN - LIMBIC	-0.00013	-5.68	<0.001	2.95E-06
	DAN - SALIENCE	-3.88E-06	-0.17	1	1
	DAN - SOMMOTOR	-4.64E-05	-2.09	0.29	1
	DAN - VIS	-4.76E-05	-2.14	0.27	1
	FPN - LIMBIC	-4.25E-05	-1.91	0.395	1
	FPN - SALIENCE	8.00E-05	3.60	0.004	0.065
	FPN - SOMMOTOR	3.75E-05	1.69	0.54	1
	FPN - VIS	3.63E-05	1.63	0.58	1
	LIMBIC - SALIENCE	0.00012	5.51	<0.001	<0.001
	LIMBIC - SOMMOTOR	8.00E-05	3.60	0.0044	0.0655
	LIMBIC - VIS	7.88E-05	3.54	0.0053	0.079
	SALIENCE - SOMMOTOR	-4.26E-05	-1.91	0.39	1
	SALIENCE - VIS	-4.37E-05	-1.97	0.36	1
**RVT**	SOMMOTOR - VIS	-1.16E-06	-0.05	1	1
	DAN - FPN	0.0015	2.36	0.17	1
	DAN - LIMBIC	0.0032	4.93	<0.001	<0.001
	DAN - SALIENCE	-0.0002	-0.31	1	1
	DAN - SOMMOTOR	0.00094	1.47	0.68	1
	DAN - VIS	0.00034	0.53	0.995	1
	FPN - LIMBIC	0.0016	2.57	0.11	1
	FPN - SALIENCE	-0.0017	-2.68	0.08	1
	FPN - SOMMOTOR	-0.00057	-0.89	0.95	1
	FPN - VIS	-0.0012	-1.83	0.445	1
	LIMBIC - SALIENCE	-0.0034	-5.24	<0.001	<0.001
	LIMBIC - SOMMOTOR	-0.0022	-3.46	0.00715	0.11
	LIMBIC - VIS	-0.0028	-4.40	<0.001	0.0024
	SALIENCE - SOMMOTOR	0.0011	1.78	0.48	1
	SALIENCE - VIS	0.00054	0.84	0.96	1
**LF-HRV**	SOMMOTOR - VIS	-0.0006	-0.94	0.94	1

Table S8. Pairwise comparisons of strength of the slope of arousal indices predicting network-network connectivity. RVT: respiration volume per unit time; LF-HRV: Percent of heart rate variability in low frequency range. Data based on n = 462 from the Human Connectome Project.

Table S9

Model term	num Df	den Df	F	p
RVT	1	13453.62	3.48E-05	1
brain state	7	3417.26	3184.787	0
session	1	10217.15	5.94E-06	1
phase	1	10091.25	2.84E-06	1
sex	1	3420.02	3.10E-06	1
age	1	3438.07	1.72E-05	1
brain state x RVT	7	13456.6	23.76	<0.001
brain state x sex	7	3420.39	14.45	<0.001
brain state x age	7	3438.48	12.18	<0.001
HF-HRV	1	12222.63	2.64E-06	1
brain state	7	3420.29	3110.18	<0.001
session	1	10233.52	4.45E-06	1
phase	1	10078.81	3.80E-06	1
sex	1	3670.76	2.61E-06	1
age	1	3415.26	9.08E-06	1
brain state x HF-HRV	7	12246.98	4.88	<0.001
brain state x sex	7	3670.54	10.37	<0.001
brain state x age	7	3415.63	11.18	<0.001
LF-HRV	1	13192.01	7.11E-06	1
brain state	7	3419.75	3158.89	<0.001
session	1	10212.32	5.17E-06	1
phase	1	10086.39	4.01E-06	1
sex	1	3531.55	7.05E-07	1
age	1	3416.20	1.03E-05	1
brain state x LF-HRV	7	13196.18	32.64	<0.001
brain state x sex	7	3531.93	9.79	<0.001
brain state x age	7	3416.66	11.22	<0.001

Table S9. Three model results for each alternate indices of arousal in predicting network temporal dynamics. All physiology measures (RVT, HF-HRV, LF-HRV) and age were demeaned. RVT: respiration volume per unit time; HF-HRV: Percent of heart rate variability in high frequency range; LF-HRV: Percent of heart rate variability in low frequency range. Data based on n = 462 from the Human Connectome Project.

Table S10.

**Model Term**	**num Df**	**den Df**	**F**	**p**
Drug	2	695.48	4.17	0.016
Network	5	321.59	173.07	p<0.001
sLFO	1	668.94	119.45	p<0.001
age	1	56.17	5.65	0.021
Sex	1	56.35	0.67	0.415
Drug:Network	10	726.46	1.25	0.255
Drug:sLFO	2	715.64	2.93	0.054
Network:sLFO	5	790.39	5.865	p<0.001
Drug:Network:sLFO	10	899.99	0.88	0.55

Table S10. Pharmacological manipulation sample DMN-network main model results for DMN x 6 functional networks (n = 59). Den df approximated via S-method. Network term includes: DMN-FPN, DMN-Limbic, DMN-DAN, DMN-SN, DMN-SMO, and DMN-VIS. sLFO was demeaned in the model.

Table S11.

Model Term	num Df	den Df	F	Pr(>F)	NETWORK
sLFO	1	168.98	39.36	<0.001	DMN-DAN
drug	2	121.72	0.91	0.41	DMN-DAN
age	1	55.90	4.28	0.04	DMN-DAN
sex	1	56.23	0.64	0.43	DMN-DAN
sLFO	1	170.22	51.70	<0.001	DMN-FPN
drug	2	121.22	1.64	0.20	DMN-FPN
age	1	55.57	3.35	0.07	DMN-FPN
sex	1	55.90	0.12	0.73	DMN-FPN
sLFO	1	170.98	4.52	0.03	DMN-LIMBIC
drug	2	121.18	0.39	0.68	DMN-LIMBIC
age	1	55.98	2.18	0.15	DMN-LIMBIC
sex	1	56.29	0.39	0.54	DMN-LIMBIC
sLFO	1	169.98	49.15	<0.001	DMN-SN
drug	2	121.13	1.34	0.27	DMN-SN
age	1	55.43	6.30	0.02	DMN-SN
sex	1	55.76	0.70	0.41	DMN-SN
sLFO	1	171.00	25.58	<0.001	DMN-SMN
drug	2	121.19	2.05	0.13	DMN-SMN
age	1	55.91	3.46	0.07	DMN-SMN
sex	1	56.23	1.40	0.24	DMN-SMN
sLFO	1	145.33	13.06	<0.001	DMN-VIS
drug	2	119.56	5.38	0.01	DMN-VIS
age	1	52.74	8.82	0.004	DMN-VIS
sex	1	53.08	2.81	0.10	DMN-VIS

Table S11. Each network-network connectivity by sLFO model controlling for drug, age, and sex with raw uncorrected p values (n = 59).

Table S12

Model Term	num Df	den Df	F	p
sLFO	1	1235.96	2.71E-12	1
brain state	7	479.76	296.7	p<0.001
Drug	2	970.60	3.10E-29	1
sLFO:brain state	7	1235.96	28.875	p<0.001
sLFO:Drug	2	1174.73	1.21E-30	1
brain state:Drug	14	970.60	3.74	p<0.001
brain state:age	8	443.90	1.97	0.049
brain state:Sex	8	446.9	0.44	0.90
sLFO:brain state:Drug	14	1174.73	1.48	0.11

Table S12. Pharmacological manipulation sample (n = 59) main model results for percentage time in eight brain states. Den df approximated via S-method. Brain state term includes: FI-DMN, FPN, DMN, DAN, SN-1, SMO, SM-DMN, SN-2. Both sLFO and age were demeaned.

Table S13.

Brain state	variable	num Df	den Df	F	p	p (corrected)
**FI-DMN**	sLFO	1	158.77	1.81	0.18	1
	Drug	2	122.53	0.52	0.59	
	Age	1	55.88	3.17	0.08	
	Sex	1	56.23	0.67	0.42	
**FPN**	sLFO	1	150.35	33.35	<0.001	<0.001
	Drug	2	121.74	3.58	0.03	
	Age	1	54.82	0.03	0.88	
	Sex	1	55.17	0.00	0.98	
**DMN**	sLFO	1	156.87	52.61	<0.001	<0.001
	Drug	2	121.62	4.30	0.02	
	Age	1	54.85	0.07	0.79	
	Sex	1	55.21	0.19	0.67	
**DAN**	sLFO	1	162.61	42.42	<0.001	<0.001
	Drug	2	121.08	4.47	0.01	
	Age	1	54.52	0.90	0.35	
	Sex	1	54.86	0.04	0.85	
**SN-1**	sLFO	1	163.71	20.50	<0.001	<0.001
	Drug	2	122.29	1.35	0.26	
	Age	1	55.86	0.42	0.52	
	Sex	1	56.20	0.62	0.43	
**SMO**	sLFO	1	164.70	0.73	0.40	1
	Drug	2	122.11	1.73	0.18	
	Age	1	55.72	2.78	0.10	
	Sex	1	56.07	0.06	0.81	
**SM-DMN**	sLFO	1	134.79	2.58	0.11	0.884
	Drug	2	121.98	5.55	0.00	
	Age	1	54.93	3.89	0.05	
	Sex	1	55.27	1.76	0.19	
**SN-2**	sLFO	1	162.62	16.58	<0.001	<0.001
	Drug	2	122.23	2.00	0.14	
	Age	1	55.73	7.23	0.01	
	Sex	1	56.08	0.34	0.56	

Table S13. Each brain state model testing relationship between sLFO and percentage of time in state while controlling for drug, age, and sex (n = 59). Raw uncorrected p values provided for all terms and Bonferroni corrected p values provided for sLFO term (N comparisons = 8).

Figure S4.

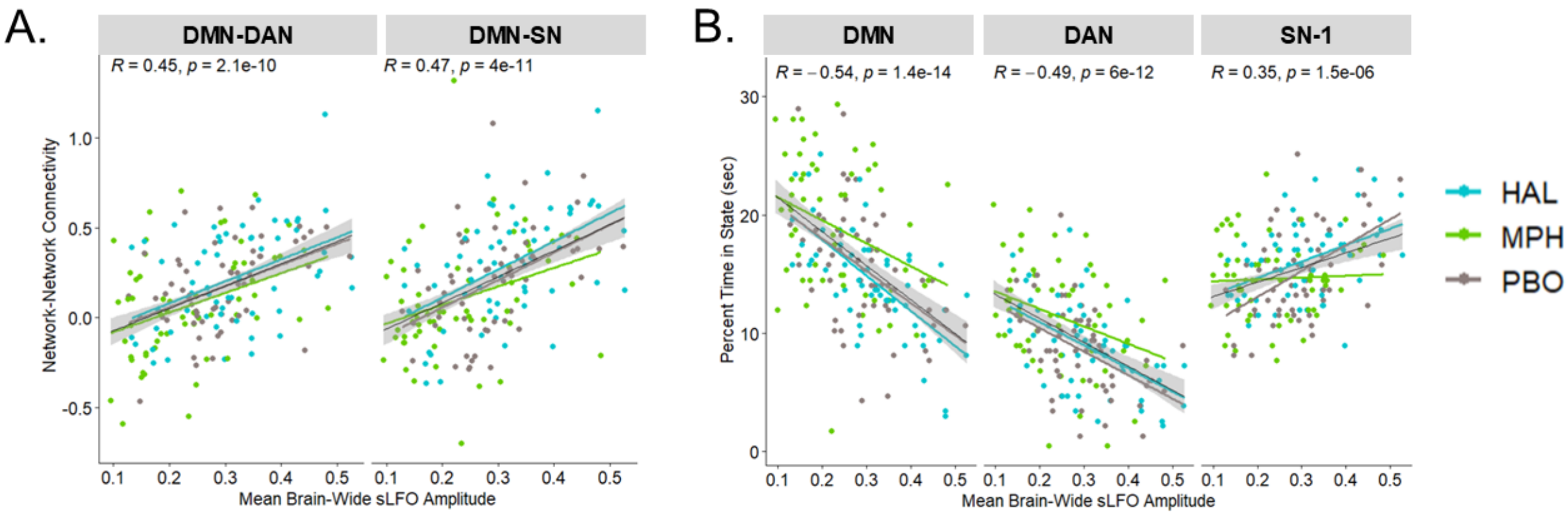

Figure S4. A. Plots of the relationship between mean sLFO amplitude and DMN-DAN and DMN-SN connectivity, under each pharmacological condition (n = 59 individuals). B. Plots of the relationship between mean sLFO amplitude and time spent in the DMN, DAN, and SN, under each pharmacological condition. DMN: Default Mode Network; DAN: Dorsal Attention Network; SN: Salience Network; HAL: haloperidol; MPH: methylphenidate; PBO: placebo.

Figure S5.

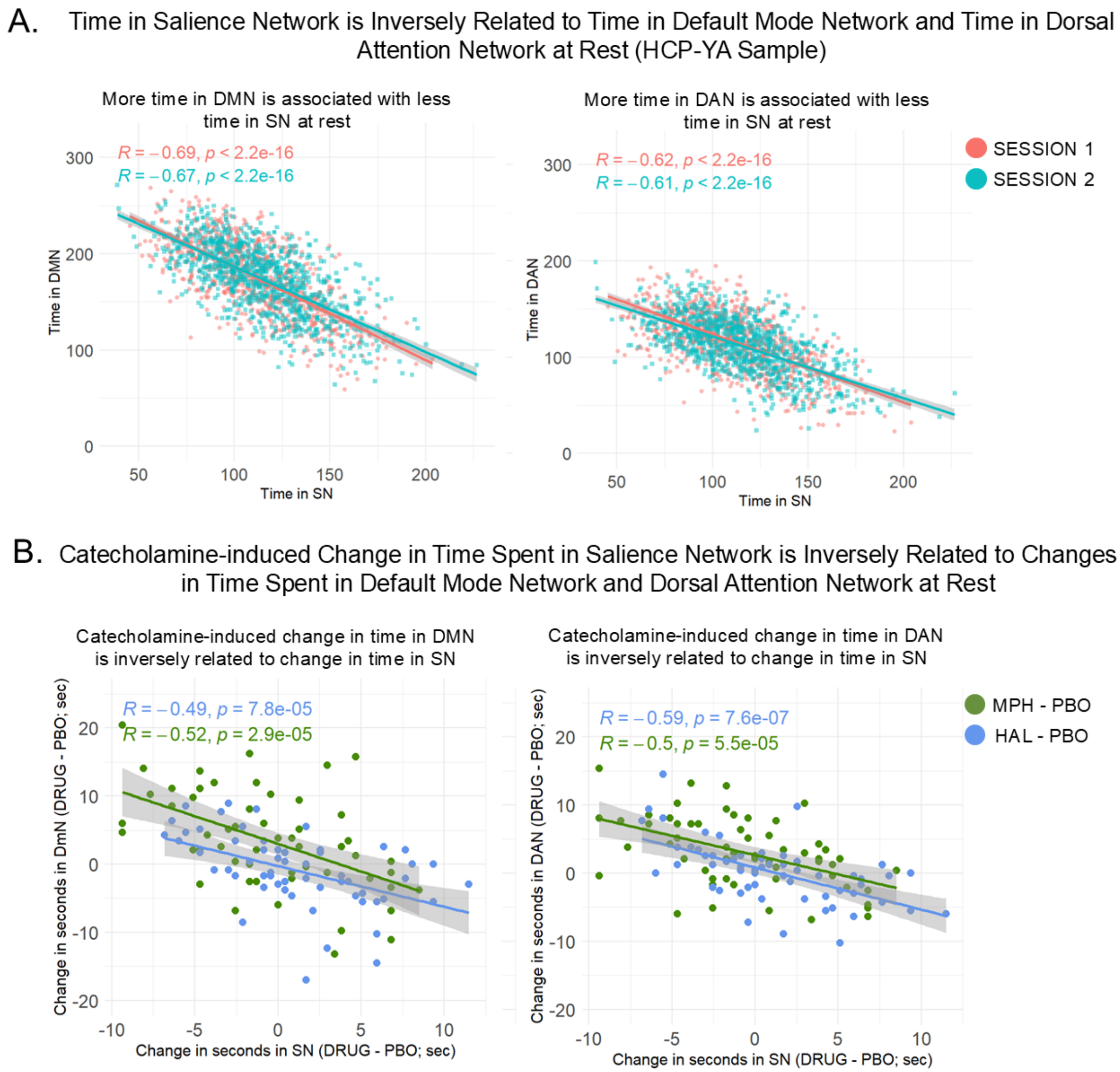

Figure S5. Time in salience network at rest is inversely related to time in the default mode network and dorsal attention network at rest. A. Relationships between time spent in SN and DMN (A) and DAN (B) at rest in HCP-YA sample (n = 462). B. Catecholamine-induced changes in time spent in SN is inversely related to catecholamine-induced changes in time spent in the DMN (A) and DAN (B) at rest in pharmacological manipulation sample (n = 59). SN: Salience Network; DMN: Default Mode Network; DAN: Dorsal Attention Network; MPH: Methylphenidate; HAL: Haloperidol; PBO: Placebo.

Supplemental Methods

Figure S6.

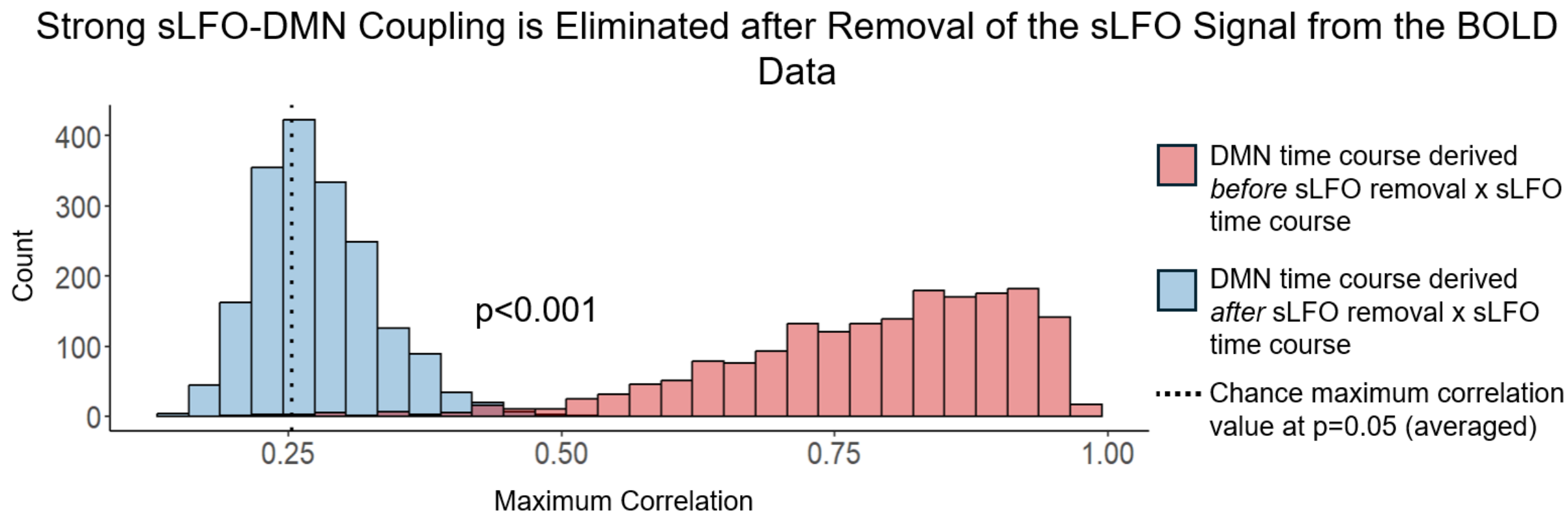

Figure S6. Strong sLFO-DMN coupling is eliminated after removal of the sLFO signal from the BOLD data. Histogram depicts distribution of with-in subject correlations (R values) of DMN time course and sLFO time course before sLFO signal removal (red) and after (blue) ($n = 462 \times 4$ sessions). Paired t-test (on z-transformed R-values) revealed significant difference ($p<0.001$) in R-values before and after sLFO signal removal.

Figure S7.

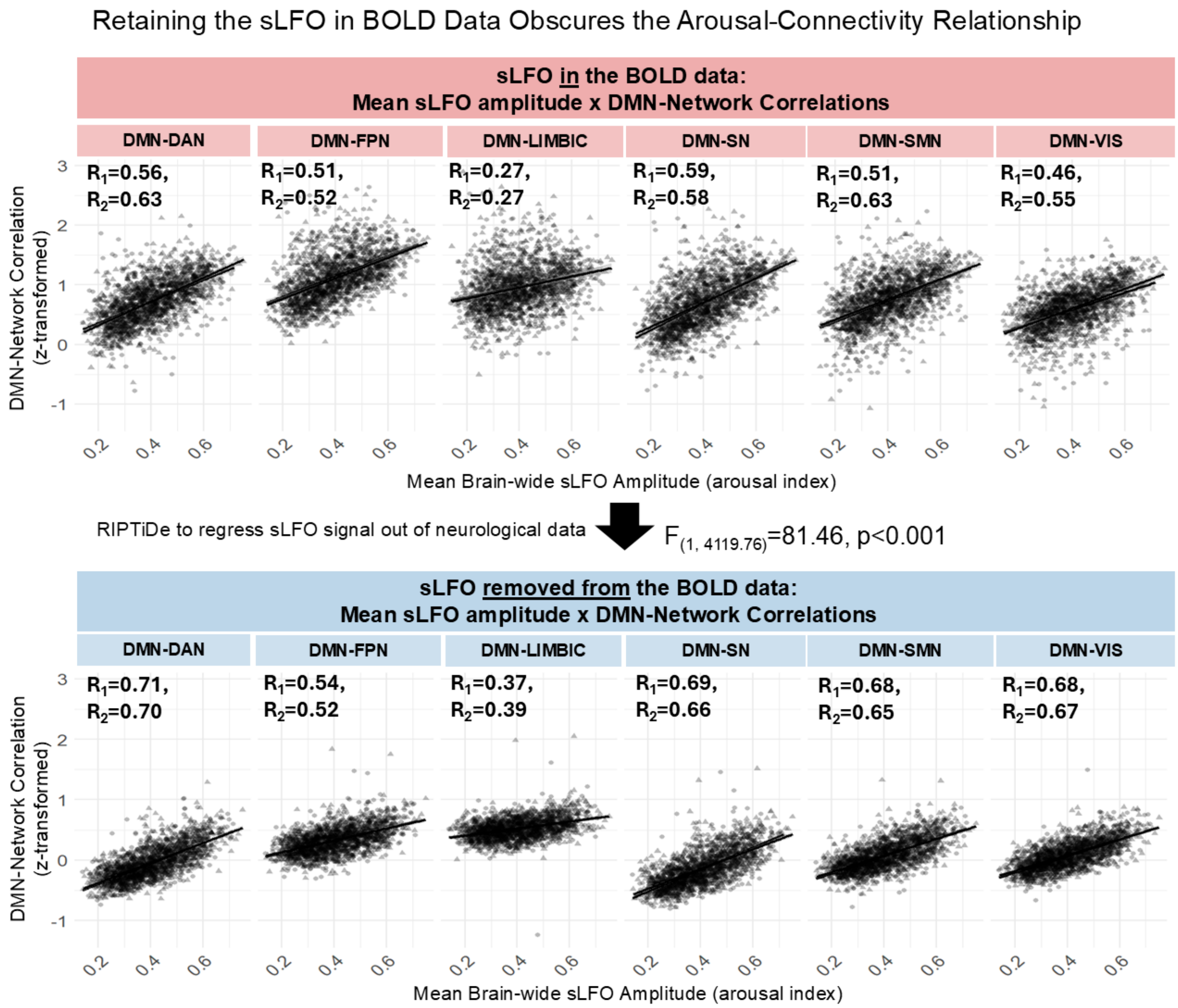

Figure S7. Removing the sLFO from BOLD data clarifies the arousal-connectivity relationship (n = 462). Top graph (red) depicts relationship between mean sLFO amplitude and DMN-network connectivity values before the sLFO signal was removed from BOLD data. Bottom graph (blue) depicts relationship between mean sLFO amplitude and DMN-network connectivity after the sLFO signal was removed from BOLD data (as seen in main Figure 1B). Note that the mean sLFO amplitude does not change from the red to blue plots; only the derived DMN-network connectivity values change, based on whether the temporal sLFO was removed from the BOLD data used to derive connectivity values. There was a significant mean sLFO amplitude x data cleaning interaction when predicting DMN-network connectivity, which indicated that the mean sLFO amplitude can significantly better explain DMN-network connectivity after temporal sLFO removal compared to before temporal sLFO removal ($F_{(1, 4119.76)} = 81.41$, $p<0.001$). We interpret this to lend confidence that shared variance between the BOLD signal and sLFO signal does not drive the robust arousal-connectivity relationships identified here.
