# Supplementary material for: Physiological arousal as a predominant source of individual differences in functional brain networks
Source: Commun Biol. 2026 Jul 10;9:1446. doi: 10.1038/s42003-026-10624-y (PMC13728107; doi:10.1038/s42003-026-10624-y)
Supplement: Supplementary file 2 [file 42003_2026_10624_MOESM2_ESM.pdf]

Description of Additional Supplementary Files

File name: Supplementary Data 1

Description: The source data behind the graphs in the paper
