# Supplementary material for: Physiological arousal as a predominant source of individual differences in functional brain networks
Source: Commun Biol. 2026 Jul 10;9:1446. doi: 10.1038/s42003-026-10624-y (PMC13728107; doi:10.1038/s42003-026-10624-y)
Supplement: Supplementary file 3 [file 42003_2026_10624_MOESM4_ESM.pdf]

nature portfolio

Corresponding author(s): Amy C. Janes

Last updated by author(s): Jun 10, 2026

Reporting Summary

Nature Portfolio wishes to improve the reproducibility of the work that we publish. This form provides structure for consistency and transparency in reporting. For further information on Nature Portfolio policies, see our Editorial Policies and the Editorial Policy Checklist.

Statistics

For all statistical analyses, confirm that the following items are present in the figure legend, table legend, main text, or Methods section.

n/a	Confirmed	
☐	☒	The exact sample size (*n*) for each experimental group/condition, given as a discrete number and unit of measurement
☐	☒	A statement on whether measurements were taken from distinct samples or whether the same sample was measured repeatedly
☐	☒	The statistical test(s) used AND whether they are one- or two-sided<br>*Only common tests should be described solely by name; describe more complex techniques in the Methods section.*
☐	☒	A description of all covariates tested
☐	☒	A description of any assumptions or corrections, such as tests of normality and adjustment for multiple comparisons
☐	☒	A full description of the statistical parameters including central tendency (e.g. means) or other basic estimates (e.g. regression coefficient) AND variation (e.g. standard deviation) or associated estimates of uncertainty (e.g. confidence intervals)
☐	☒	For null hypothesis testing, the test statistic (e.g. *F*, *t*, *r*) with confidence intervals, effect sizes, degrees of freedom and *P* value noted<br>*Give P values as exact values whenever suitable.*
☐	☒	For Bayesian analysis, information on the choice of priors and Markov chain Monte Carlo settings
☐	☒	For hierarchical and complex designs, identification of the appropriate level for tests and full reporting of outcomes
☐	☒	Estimates of effect sizes (e.g. Cohen's *d*, Pearson's *r*), indicating how they were calculated

Our web collection on statistics for biologists contains articles on many of the points above.

Software and code

Policy information about availability of computer code

Data collection	N/A: Data was collected using standard fMRI sequences
Data analysis	The systemic low-frequency oscillation (sLFO) signal was derived using rapidtide: https://github.com/bbfrederick/rapidtide<br>Neuroimaging analyses were conducted using FSL: https://fsl.fmrib.ox.ac.uk/fsl/docs/install/index.html, AFNI: https://afni.nimh.nih.gov/, and R: https://www.r-project.org/

For manuscripts utilizing custom algorithms or software that are central to the research but not yet described in published literature, software must be made available to editors and reviewers. We strongly encourage code deposition in a community repository (e.g. GitHub). See the Nature Portfolio guidelines for submitting code & software for further information.

Data

Policy information about availability of data

All manuscripts must include a data availability statement. This statement should provide the following information, where applicable:

- Accession codes, unique identifiers, or web links for publicly available datasets
- A description of any restrictions on data availability
- For clinical datasets or third party data, please ensure that the statement adheres to our policy

Data collected at the National Institute on Drug Abuse (NIDA) can be made available per request and after obtaining a data transfer agreement. Data from the Human Connectome Project (HCP) is publicly available.

Research involving human participants, their data, or biological material

Policy information about studies with human participants or human data. See also policy information about sex, gender (identity/presentation), and sexual orientation and race, ethnicity and racism.

Reporting on sex and gender	Sex differences were not studied in this work as some of the cohorts were not powered to assess sex differences. However, sex was controlled for in each analysis and this is presented in the supplement. See table S4.
Reporting on race, ethnicity, or other socially relevant groupings	Race, ethnicity, or other socially relevant groupings were not studied in this work.
Population characteristics	See above
Recruitment	All participants were recruited from the general population using standard recruitment methods such as fliers and online adds. Recruitment was done in the community proximal to each study, either in the general community around NIDA (Baltimore and surrounding locations) or proximal to the HCP site locations.
Ethics oversight	For the Acute Drug Administration Cohort: All procedures were completed at the National Institute on Drug Abuse and were approved by the institutional review board of the National Institutes of Health.<br><br>HCP Cohort: All data were coded and no longer considered human subject's data. Initial data collection was approved by the Institutional Review Board at Washington University in St. Louis.

Note that full information on the approval of the study protocol must also be provided in the manuscript.

Field-specific reporting

Please select the one below that is the best fit for your research. If you are not sure, read the appropriate sections before making your selection.

☐ Life sciences ☒ Behavioural & social sciences ☐ Ecological, evolutionary & environmental sciences

For a reference copy of the document with all sections, see nature.com/documents/nr-reporting-summary-flat.pdf

Behavioural & social sciences study design

All studies must disclose on these points even when the disclosure is negative.

Study description	All data allowed for the evaluation of the sLFO on brain network function, both connectivity and temporal dynamics.<br><br>The Acute Drug Administration Cohort allowed this work to be tested in an independent cohort and to determine the impact of pharmacological impact on these measures. The Acute Drug Administration Cohort included data collected in a randomized, double blind, cross over manner.<br><br>The HCP data set allowed this work to be tested within the same participants across two independent days.
Research sample	All participants came from the local community and were healthy volunteers.<br><br>Acute Drug Administration Cohort: N = 59 individuals<br>HCP Cohort: N = 462 individuals
Sampling strategy	Healthy volunteers from the local communities were recruited for and enrolled based on meeting safety criteria for performing neuroimaging or pharmacological research.
Data collection	Data were collected using neuroimaging techniques. In the Acute Drug Administration Cohort, study drugs were administered in a randomized, double-blind, placebo-controlled design.
Timing	Acute Drug Administration Cohort: Data collection took place at the National Institute on Drug Abuse in Baltimore, MD. Samples were collected between 8/2013 and 9/2018.<br><br>HCP Cohort: Data collection took place at Washington University in St. Louis, Missouri. Samples were collected between 8/2012 and 3/2015.
Data exclusions	Acute Drug Administration Cohort: N = 76 were allocated to intervention following eligibility assessments using inclusion/exclusion criteria detailed in the manuscript. During the study 3 were excluded- specific reasons were MRI artifact (1) and exclusionary substance use (2). Thus in addition to drop out due to non-participation, (see below) N = 67 individuals completed all sessions. N = 59 were analyzed and 8 were excluded during analysis. These exclusions included signal dropout (5), incorrect scan parameters (1), excessive movement (1) , exclusionary substance use discovered after study completion (1).<br><br>HCP Cohort: N = 744 individuals were excluded. Individuals were excluded if they were found to be ineligible for reasons noted in the

	inclusion/exclusion description of the manuscript supplement (ie, lifetime history of significant alcohol/substance use, contraindicated health/mental health condition).
Non-participation	Acute Drug Administration Cohort: N = 76 were allocated to intervention, and N = 6 of these individuals did not complete all sessions due to: lost to follow up (1), participant scheduling constraints (3), participant decision to discontinue following medication side effects (2).
Randomization	Participants in the acute drug administration cohort were administered drug or placebo in a randomized, double blind, placebo controlled manner.

Reporting for specific materials, systems and methods

We require information from authors about some types of materials, experimental systems and methods used in many studies. Here, indicate whether each material, system or method listed is relevant to your study. If you are not sure if a list item applies to your research, read the appropriate section before selecting a response.

Materials & experimental systems

n/a	Involved in the study
☒	☐ Antibodies
☒	☐ Eukaryotic cell lines
☒	☐ Palaeontology and archaeology
☒	☐ Animals and other organisms
☐	☒ Clinical data
☒	☐ Dual use research of concern
☒	☐ Plants

Methods

n/a	Involved in the study
☒	☐ ChIP-seq
☒	☐ Flow cytometry
☐	☒ MRI-based neuroimaging

Clinical data

Policy information about clinical studies

All manuscripts should comply with the ICMJE guidelines for publication of clinical research and a completed CONSORT checklist must be included with all submissions.

Clinical trial registration	Acute Drug Administration Cohort: NCT01924468<br>HCP Cohort: N/A
Study protocol	Acute Drug Administration Cohort: Please contact the last author as this can be received from NIDA after a data transfer agreement is in place.<br>HCP Cohort: Data from the Human Connectome Project is publicly available on the open access Connectome database (https://db.humanconnectome.org/app/template/Login.vm), which can be accessed after signing a data use agreement.
Data collection	Acute Drug Administration Cohort-- The study: “Brain Networks and Addiction Susceptibility NCT01924468” does not have a phase designation. Here, our study used data following pharmacological manipulation in healthy individuals which may be classified as intervention. We thus have completed the CONSORT Checklist for this study where applicable.<br><br>HCP Cohort: The study using data from the Human Connectome Project was de-identified at time of data accrual and does not classify as a Clinical Trial. Thus, the CONSORT Checklist does not apply.
Outcomes	Outcomes focused on the relationship between the sLFO, which is a novel analysis method defined after the data were collected. However, the questions investigated focused on the focus of the nitial studies.<br><br>Acute Drug Administration Cohort: The primary focus of this study was to investigate the neurobiological impact of medications on the brain.<br><br>HCP Cohort: to map neural pathways and understand structure, function, and behavior. Here, we evaluate the relationship of the sLFO on these metrics.<br><br>While the focus on the sLFO signal in the current paper is novel, the initial goals of each study match this focus.

Plants

Seed stocks	N/A
Novel plant genotypes	N/A
Authentication	N/A

Magnetic resonance imaging

Experimental design

Design type	Both Cohorts: resting state fmri
Design specifications	HCP: Cross sectional, two independent scans allowing for within subjects comparison<br>Acute Drug Administration Cohort: drugs administered prior to scanning such that data acquisition took place at peak drug effect. Drugs administered in a placebo controlled, double blind, random manner
Behavioral performance measures	N/A

Acquisition

Imaging type(s)	Functional MRI
Field strength	3 Tesla
Sequence & imaging parameters	Acute Drug Administration Cohort: Data were acquired on a Siemens Trio 3T scanner (Erlangen, Germany) using a 12 channel head coil. The total scanning session included an 8-minute resting-state acquisition prior to the three, approximately 11-minute blocks of the Multi-Source Interference Task (MSIT). Functional scan acquisition used the following parameters: TR = 2 s, TE = 27 ms, flip angle = 78o, resolution = 3.4375 mm x 3.4375 mm x 4 mm. Following the resting state scan, structural images using were acquired using the following parameters: TR = 1.9 s, TE= 3.51 ms, flip angle = 9o, resolution: 1.0 x 1.0 x 1.0 mm.<br><br>HCP Cohort: The opensource HCP minimal preprocessed resting-state fMRI data was used in this work. Data were acquired on a Siemens 3T Skyra scanner (Erlangen, Germany) modified to achieve a maximum gradient strength of 100 mT m-1 using a 32-channel head coil. Participants underwent two resting-state scans occurring on different days. Data were acquired across four, 14.4-minute resting state runs—two runs were completed during one visit and two in a separate visit. Within each session, oblique axial acquisitions alternated between phase encoding in a right-to-left (RL) direction during one run and phase encoding in a left-to-right (LR) direction during the other run. Functional scan acquisition used the following parameters: TR = 0.72 s, TE = 33.1 ms, flip angle = 52o, field of view = 280 x 180 mm2, matrix = 140 x 90, echo spacing = 0.58 ms, bandwidth = 2,290 Hz px-1. Slice thickness was set to 2.0 mm, 72 slices and 2.0 mm isotropic voxels, multiband acceleration factor = 8.
Area of acquisition	Whole Brain

Diffusion MRI ☐ Used ☐ Not used

Preprocessing

Preprocessing software	Acute Drug Administration Cohort: To preprocess the data, raw MRI DICOM data were converted to the standard BIDS structure with bidskit (https://github.com/jmtyszka/bidskit). Data were preprocessed using fMRIPrep (v.22.1.0, https://fmriprep.org/en/22.1.0/), which included discarding the first 2 volumes to account for the T1 non-equilibrium, slice timing correction, volume registration, head motion estimation, and spatial normalization to MNI space.<br><br>HCP Cohort: The opensource HCP minimal preprocessed resting-state fMRI data was used in this work.
Normalization	Acute Drug Administration Cohort: Standard FSL linear and non linear normalization<br>HCP Cohort: The opensource HCP minimal preprocessed resting-state fMRI data was used in this work.
Normalization template	All data were normalized to MNI space.
Noise and artifact removal	Acute Drug Administration Cohort: motion correction, FIX<br>HCP Cohort: used the minimal preprocessed resting state

	Both data sets: rapidtide was used to remove the sLFO as this was the focus of the paper
Volume censoring	N/A.

Statistical modeling & inference

Model type and settings	No tasks were conducted. All statistics were conducted outside of fMRI packages to evaluate connectivity and dynamics.
Effect(s) tested	Functional connectivity and temporal dynamics relationship with the sLFO was evaluated

Specify type of analysis: ☐ Whole brain ☐ ROI-based ☐ Both

Statistic type for inference (See Eklund et al. 2016)	N/A
Correction	Corrections not done within fMRI packages

Models & analysis

n/a	Involved in the study
☐	☒ Functional and/or effective connectivity
☒	☐ Graph analysis
☒	☐ Multivariate modeling or predictive analysis

Functional and/or effective connectivity	We derived the Fisher r-to-z transformed Pearson's correlation coefficient (r) between each pair of network-network time courses (21 correlations per subject). For the Acute Drug Administration Cohort, r-to-z correlation values were derived under each drug condition. For the HCP Cohort, r-to-z correlation values were derived for each scan session and each phase encode direction (four total).
